# Microbial Protein Binding to gC1qR Drives PLA2G1B-Induced CD4 T-Cell Anergy

**DOI:** 10.3389/fimmu.2022.824746

**Published:** 2022-03-22

**Authors:** Julien Pothlichet, Annalisa Meola, Florence Bugault, Louise Jeammet, Anne G. Savitt, Berhane Ghebrehiwet, Lhousseine Touqui, Philippe Pouletty, Frédéric Fiore, Alain Sauvanet, Jacques Thèze

**Affiliations:** ^1^ DIACCURATE, Paris, France; ^2^ Division of Rheumatology, Allergy, and Clinical Immunology, Department of Medicine, SUNY Stony Brook, Stony Brook, NY, United States; ^3^ Cystic Fibrosis and Bronchial Diseases team - INSERM U938, Institut Pasteur, Paris, France; ^4^ Centre de Recherche Saint-Antoine (CRSA) - INSERM UMRS938, Sorbonne Université, Paris, France; ^5^ Truffle Capital, Paris, France; ^6^ Centre d’Immunophénomique, Aix Marseille Université, INSERM, CNRS, Marseille, France; ^7^ Service de Chirurgie Hépatobiliaire et Pancréatique - Department of HBP Surgery, Hôpital Beaujon - University of Paris, Clichy, France

**Keywords:** HIV, PLA2G1B, CD4 T cell, gC1qR, HCV, staphylococcus aureus, porphyromonas gingivalis, infectious disease

## Abstract

The origin of the impaired CD4 T-cell response and immunodeficiency of HIV-infected patients is still only partially understood. We recently demonstrated that PLA2G1B phospholipase synergizes with the HIV gp41 envelope protein in HIV viremic plasma to induce large abnormal membrane microdomains (aMMDs) that trap and inactivate physiological receptors, such as those for IL-7. However, the mechanism of regulation of PLA2G1B activity by the cofactor gp41 is not known. Here, we developed an assay to directly follow PLA2G1B enzymatic activity on CD4 T-cell membranes. We demonstrated that gp41 directly binds to PLA2G1B and increases PLA2G1B enzymatic activity on CD4 membrane. Furthermore, we show that the conserved 3S sequence of gp41, known to bind to the innate sensor gC1qR, increases PLA2G1B activity in a gC1qR-dependent manner using gC1qR KO cells. The critical role of the 3S motif and gC1qR in the inhibition of CD4 T-cell function by the PLA2G1B/cofactor system in HIV-infected patients led us to screen additional microbial proteins for 3S-like motifs and to study other proteins known to bind to the gC1qR to further investigate the role of the PLA2G1B/cofactor system in other infectious diseases and carcinogenesis. We have thus extended the PLA2G1B/cofactor system to HCV and *Staphylococcus aureus* infections and additional pathologies where microbial proteins with 3S-like motifs also increase PLA2G1B enzymatic activity. Notably, the bacteria *Porphyromonas gingivalis*, which is associated with pancreatic ductal adenocarcinoma (PDAC), encodes such a cofactor protein and increased PLA2G1B activity in PDAC patient plasma inhibits the CD4 response to IL-7. Our findings identify PLA2G1B/cofactor system as a CD4 T-cell inhibitor. It involves the gC1qR and disease-specific cofactors which are gC1qR-binding proteins that can contain 3S-like motifs. This mechanism involved in HIV-1 immunodeficiency could play a role in pancreatic cancer and several other diseases. These observations suggest that the PLA2G1B/cofactor system is a general CD4 T-cell inhibitor and pave the way for further studies to better understand the role of CD4 T-cell anergy in infectious diseases and tumor escape.

## Introduction

CD4 T cells orchestrate efficient antimicrobial and antitumoral immunity. Impairment of CD4 T cells function plays a critical role in several diseases. One of the most highly studied is the severe immunodeficiency of HIV-infected patients. We recently showed that more than 80% of CD4 T cells from HIV-infected patients exhibited morphological anomalies characterized by numerous large abnormal membrane microdomains (aMMDs) that trap and inactivate physiological receptors, resulting in CD4 T-cell unresponsiveness (anergy) (1). Such aMMD-bearing cells were named “Bumpy T cells”, due to their appearance upon microscopic observation. We identified phospholipase A2 group 1B (PLA2G1B) as the key molecule responsible for the formation of aMMDs in HIV viremic patients (VP) plasma. Treatments of CD4 T cells isolated from healthy donors (HD) with purified recombinant PLA2G1B induced Bumpy T cells, similarly to HIV VP plasma. HD plasma had no effect. Immunodepletion experiments, as well as experiments with an antibody that inhibits the enzymatic activity of PLA2G1B, confirmed that the PLA2G1B molecule is the active component in the plasma of HIV-infected VP that inhibits the CD4 T-cell response to IL-7, IL-2, and IL-4. These large aMMDs were shown to trap the IL-7R alpha chain and the gamma-c chain which is common to IL-7, IL-2, and IL-4 receptors. These cytokine receptors lose their function when embedded in such aMMDs. Consequently, the Jak/STAT pathway was not functional and IL-7-induced phospho-STAT5 nuclear translocation (pSTAT5-NT) was inhibited and used as a standard assay to follow PLA2G1B activity (1).

PLA2G1B was initially named pancreatic PLA2 due to its primary production in pancreatic tissue. Two forms of PLA2G1B are present in the pancreas, the intestinal tract, and in human plasma (1). It is expressed as an inactive precursor called proPLA2G1B, which contains a propeptide in the N-terminal part that masks the active site of PLA2G1B and blocks the access of the lipid substrate to the catalytic site of the protein (2). Upon digestion by a trypsin protease, the propeptide is removed to generate the active form of PLA2G1B (active PLA2G1B). Immunohistochemistry analysis of normal human pancreatic tissue has shown that active PLA2G1B is present in the exocrine component of the pancreas but not in the endocrine component, whereas proPLA2G1B is also present in the endocrine component (1). Active PLA2G1B was initially described for its role in the intestinal absorption of lipids before our recent demonstration of its role in CD4 T-cell anergy and CD4 T-cell lymphopenia in HIV-infected patients (1).

The inhibitory activity of PLA2G1B that induces the Bumpy phenotype was observed in the plasma of HIV VP but not that of HD, ART-treated patients (ART), or HIV controllers (HIC). The quantification of active PLA2G1B by ELISA showed the protein concentration to be similar in the plasma of VP, ART and HIC, with the median only 1.4-fold higher in VP than in HD plasma. However, PLA2G1B activity was only observed in VP plasma. Thus, we postulated that other factors present in VP plasma may boost the activity of PLA2G1B on CD4 T cells. We showed that recombinant HIV gp41 envelope protein increases PLA2G1B activity on CD4 T cells in a dose-dependent manner using the pSTAT5-NT response to IL-7 as a read-out. The activity of the gp41 cofactor depends on a sequence with three serine amino acids called 3S (3) conserved among HIV strains, as a similar increase in PLA2G1B inhibitory activity was observed with the 3S peptide alone (1).

These findings led us to propose a role for the PLA2G1B/cofactor system as a negative regulator of CD4 T-cell function. We have further shown that both components of the system are essential for its activity in the plasma of VP. PLA2G1B is the active component as anti-PLA2G1B Ab abrogates the inhibitory activity of VP plasma. Moreover, immunodepletion of gp41 from HIV VP plasma, using a polyclonal anti-gp41 antibody or an anti-gp41 antibody directed against the 3S sequence, almost completely abrogates the inhibitory activity of VP plasma on CD4 T cells. In this system, PLA2G1B activity is regulated by gp41 (or 3S) as a cofactor that appears to target PLA2G1B to the CD4 T-cell surface. The PLA2G1B/gp41 pair constitutes a mechanism of immune dysfunction and a compelling target for boosting immune responses in HIV-infected patients. However, the precise mode of action of the effect of the gp41/3S cofactor on the membrane is not known (1).

Here, we first confirmed our previous observations that the inhibitory activity of VP plasma on the pSTAT5-NT response to IL-7 is due to the enzymatic activity of PLA2G1B using several PLA2 inhibitors and the anti-PLA2G1B mAb that we developed to inhibit PLA2G1B. We developed an assay to directly investigate the effect of gp41 protein on the enzymatic activity of PLA2G1B on the cell surface membrane of CD4 T cells based on the labelling of primary human CD4 T cells with tritiated arachidonic acid ([3H]AA). Thus, the enzymatic activity of PLA2G1B can be measured by quantifying the radioactivity in the cell supernatant released from the membrane of the radiolabeled CD4 T cells. We show that [3H]AA is released in a PLA2G1B dose-dependent manner and that this activity correlates with the inhibition of pSTAT5-NT. Moreover, the enzymatic activity of PLA2G1B on CD4 T cells increases in a gp41 dose-dependent manner, confirming the cofactor effect of gp41, which directly enhances PLA2G1B activity on the membranes of CD4 T cells.

The gp41/3S sequence binds to the receptor for the globular head of complement component C1q, the gC1q receptor (gC1qR) (4, 5). Notably, we showed that the gp41/3S peptide increases PLA2G1B activity on WT but not gC1qR KO cells, showing that the stimulation of PLA2G1B enzymatic activity by 3S is gC1qR-dependent. The gC1qR has been shown to bind to several microbial proteins (6). Thus, we postulated that the regulation of PLA2G1B may not be restricted to the case of HIV infection but shared by other infections. In support of this hypothesis, the HCV core and *Staphylococcus aureus* protein A, two proteins that bind to the gC1qR, also increase the enzymatic activity of PLA2G1B on the CD4 T-cell membrane. Moreover, we performed in-silico screening for 3S-like motifs in protein databases to identify additional gC1qR binding proteins that could also act as PLA2G1B cofactors. We identified 42 candidate proteins, including several proteins encoded by human pathogens. Among them, one was derived from *Porphyromonas gingivalis* (*P. gingivalis*), which is responsible for periodontal infections and has been shown to be associated with a higher risk of pancreatic cancer (7–9). Strikingly, the *P. gingivalis* 3S-like peptide also increases the enzymatic activity of PLA2G1B. We thus tested the plasma of patients with pancreatic ductal adenocarcinoma (PDAC) for the presence of inhibitory PLA2G1B activity that impairs the CD4 T-cell response to IL-7. As shown for HIV, PDAC plasma contains an activity that inhibits pSTAT5-NT in CD4 T cells. PLA2G1B is involved in inhibitory effect of PDAC plasma, as it was partially blocked by a specific anti-PLA2G1B mAb.

Finally, our findings identify a mechanism of inhibition of CD4 T-cell function through the PLA2G1B/cofactor system. PLA2G1B activity is enhanced by cofactors binding to gC1qR. These cofactors are proteins with 3S-like motifs and proteins that bind to the innate sensor gC1qR. This mechanism involved in HIV-1 immunodeficiency is likely to play a role in several diseases.

## Materials and Methods

### Recombinant Proteins and Peptides

Human PLA2G1B was produced in *E. coli* (gift of Gerard Lambeau, purity > 98%) or CHO-S (purity > 98%, Merck or GTP). Human proPLA2G1B, PLA2GIIA, PLA2GIID, PLA2GX, and WT and H48Q porcine PLA2G1B were produced in *E. coli* (gift of Gerard Lambeau, purity > 98%). Recombinant HIV-1 gp41 MN protein was obtained from Antibodies online (gp41 MN (565-771Delta642-725), containing a deletion to remove the transmembrane domain of the protein, ABIN2129703, lot 93-482, purity > 95%) and the 3S peptide NH2-PWNASWSNKSLDDIW-COOH, 3S-like peptide OmpA Pg NH2-SGEGGWSNGSLVDIM-COOH, and scrambled 3S NH2-WNWDSKILSDPAWNS-COOH peptide were ordered from Covalab (purity > 98%). The HCV core protein was obtained from Prospec (HCV-011, purity > 95%) in PBS buffer with 0.002% SDS and the specificity of the effect due to HCV core protein evaluated by comparison with a similar dilution of PBS-0.002% SDS. *Staphylococcus aureus* protein A was obtained from Sigma (P6031). sPLA2 inhibitors were obtained from several providers: Pentapeptide (sPLA_2_-IIA Inhibitor I, 525145, Calbiochem, ordered from VWR), Varespladib (LY315920, S1110, Selleck ordered from Euromedex), and sPLA2R1 (murine soluble receptor, 5367-PL-050, R&D Systems).

### Study Design And Human Sample Collection

The group of viremic patients included in the study of T lymphocytes and plasma consisted of patients with untreated chronic HIV-infection. These patients had never received antiretroviral drugs at the time of blood collection, their CD4 counts were > 300/mm^3^, and their viral loads > 10,000 copies/mL (ANRS EP 33). All blood samples from VP were drawn at the Hôpital Bicêtre, Paris. Blood from HD was obtained from healthy volunteers through the Etablissement Français du Sang (Centre Necker-Cabanel and St-Louis, Paris). Blood samples from PDAC patients were drawn at the Hôpital Beaujon (Clichy) or PDAC plasma samples were acquired from BIOIVT. These patients had never received treatments at the time of blood collection.

### Study Approval

The study of the effect of VP plasma on the pSTAT5-NT response of CD4 T cells was supported by the ANRS and approved by the “Comité Consultatif de Protection des Personnes dans la Recherche Médicale” under the number 05-15. All participants were adults and provided written informed consent prior to inclusion in the study.

All participants who provided PDAC plasma were adults and provided written informed consent prior to inclusion in the study. The study was registered under the number DC-2021-4516 by the Ministère de l’Enseignement Supérieur, de la Recherche et de l’Innovation.

### Purification of Human CD4 T-Lymphocytes

Venous blood was obtained from healthy volunteers through the EFS (Etablissement Français du Sang, Centre Necker-Cabanel, Paris). CD4 T-cells were purified from whole blood using the RosetteSep Human CD4+ T-cell Enrichment Cocktail (Stem Cell, 15062). This cocktail contains mouse and rat monoclonal antibodies purified from mouse ascites fluid or hybridoma culture supernatant, by affinity chromatography using protein A or Protein G Sepharose. These antibodies are bound in bispecific tetrameric antibody complexes directed against cell-surface antigens on human hematopoietic cells (CD8, CD16, CD19, CD36, CD56, CD66b, TCRγ/δ) and glycophorin A on red blood cells. The RosetteSep antibody cocktail crosslinks unwanted cells in human whole blood to multiple red blood cells, forming immunorosettes. This increases the density of unwanted cells, such that they pellet along with the free red blood cells when centrifuged through a buoyant density medium such as lymphocyte separation medium (Eurobio, CMSMSL01-01).

Whole blood was incubated with RosetteSep Human CD4+ T-cell Enrichment Cocktail at 50 µL/mL for 20 min at room temperature under gentle shaking (100 rpm), diluted with an equal volume of PBS + 2% fetal bovine serum (FBS), and mixed gently. The diluted samples were centrifuged for 20 min at 1,200 X g through lymphocytes separation medium. The enriched cells were then collected from the density medium at the plasma interface and washed twice with PBS + 2% FBS. Cells were subsequently resuspended in RPMI 1640 medium (Lonza) supplemented with 5% FBS, 50 mM HEPES pH 7.4, glutamine, penicillin, streptomycin and fungizone (complete medium), and counted using a Moxi Z mini automated cell counter (ORFLO, MXZ000). The cell suspension was adjusted to 7 x 10^6^ cells/mL and equilibrated for at least 2 h at 37°C in a 5% CO2 humidified atmosphere.

The enriched CD4-T cell population was analyzed by flow cytometry on a Cytoflex instrument (Beckman coulter). The quiescence of recovered CD4 T cells was verified by the low level of IL-2Rα (CD25). CD4 T cells were labeled with anti-Human CD3 eFluor780 (eBioscience, clone UCHT1, 47-0038-42), anti-Human CD25-PE (Biolegend, clone BC96, 302605), and anti-human CD4-PerCP (BD, clone SK3, 345770). The enriched CD4 T-cell population contains > 95% CD3+CD4+ and < 8% of CD25+.

### Phosphorylation and Nuclear Translocation of STAT5 (pSTAT5-NT)

STAT5 phosphorylation and nuclear translocation in HD CD4 T cells were analyzed by microscopy after IL-7 stimulation (2 nM), or in HD CD4 T cells incubated with plasma samples from HD (not shown here), HIV VP or PDAC patients (30 min), human PLA2G1B recombinant proteins, with or without a pretreatment (25 min at room temperature and 5 min at 37°C) with anti-PLA2G1B (14G9, previously described (1)) neutralizing mAb or control isotype (Mouse IgG1, 16-4714-85, Thermofisher) or sPLA2 inhibitors (pentapeptide, varespladib, sPLA2R1) before a 15 min of stimulation with 2 nM IL-7 (recombinant glycosylated human IL-7, Accrobio System). All cell treatments were performed at 37°C. Cell supernatants were removed and stimulation was stopped by the addition of 500 µl of a 4% paraformaldehyde solution in PBS (Fisher, PFA 32% Electron Microscopy Science, 15714) and incubation for 15 min at 37°C. Cells were then permeabilized overnight at -20°C in 500 µL of an ice-cold 90% methanol/water solution.

CD4 T cells were stained using anti-human CD4 (mouse anti-CD4 clone RPA-T4, 555344, BD Biosciences; or goat anti-CD4, AF-379-NA, R&D/Novus), followed by donkey anti-mouse-AlexaFluor488 (A21202, Thermofisher). Phosphorylation of STAT5 in response to IL-7 stimulation was then revealed by staining with rabbit anti-pSTAT5 (9359, Cell Signaling Technology) followed by goat anti-rabbit-Atto 647N (15068; Active Motif) or donkey anti-rabbit AlexaFluor555 (A31572, Life Technologies). Briefly, slides were washed twice after methanol treatment in PBS and cells were rehydrated for 15 min in PBS supplemented with 5% FBS at room temperature. Slides were labeled with primary antibodies (1/120) in 60 µl of PBS-5% FBS for 1 h, washed in PBS buffer 15 times, and washed in PBS/FBS buffer five times. Slides were then stained with secondary antibodies (1/300) for 1 h, washed five times in PBS-5% FBS buffer, rinsed 15 times in PBS, and then mounted in fresh Prolong Gold Antifade (ThermoFisher Scientific, P36930) mounting medium for confocal microscopy.

Images were acquired above the diffraction limit using an inverted laser scanning confocal microscope (LSM700, Zeiss) as previously described (1). The appearance of pSTAT5 was measured using ImageJ software. The number of cells positive for nuclear pSTAT5 among > 200 in response to cytokines was analyzed by confocal microscopy.

### PLA2G1B Enzymatic Assay on [3H] Arachidonic Acid-Labeled CD4 T Cells

Purified CD4 T-cells were incubated for 16 h at 2 x 10^6^ cells/mL with 1 µCi/mL of arachidonic acid [5,6,8,9,11,14,15-^3^H(N)] (Perkin Elmer, NET298Z250UC) in RPMI 1640 medium (Lonza) supplemented with 10% FBS, 50 mM HEPES pH 7.4, glutamine, penicillin, streptomycin and fungizone at 2 mL/well in six-well plates at 37°C in a humidified 5% CO2 atmosphere. Cells were washed twice with RPMI containing 10% FBS by centrifugation at 580 x g for 10 min at room temperature and then frozen in 90% FBS 10% DMSO at 10^7^ cells/mL/vial at -80°C. The percentage of [3H] arachidonic acid in CD4 T cells (1 minus the ratio of [3H] arachidonic acid in the CD4 T-cell supernatant (cpm/mL) relative to the total [3H] arachidonic acid in the supernatant and cells (cpm/mL)) was measured to control the cell preparation.

To test PLA2G1B activity on [3H] arachidonic acid ([3H]-AA)-labeled CD4 T lymphocytes, cells were first thawed in 10% FBS RPMI preheated to 37°C, centrifuged at 580 x g for 10 min at room temperature, washed twice in 2.5% FBS RPMI, and equilibrated in 2.5% FBS RPMI at 2 x 10^5^ CD4 T cells/400µL/well in 24-well polystyrene plates for 90 min at 37°C in a humidified 5% CO2 atmosphere.

To test the effect of H48Q and sPLA2 inhibitors on PLA2G1B activity on [3H]-AA-labeled CD4 T lymphocytes, 100 µL of recombinant WT human (hPLA2G1B) and WT and the catalytic-site mutant H48Q porcine PLA2G1B (pPLA2G1B), or medium or hPLA2G1B pretreated (25 min at room temperature and 5 min at 37°C) with sPLA2 inhibitors (pentapeptide, varespladib) or vehicle, in 2.5% FBS RPMI was added to each well and the plates incubated for 2 h.

To test the effect of viral or bacterial protein cofactors on PLA2G1B activity on [3H]-AA-labeled CD4 T lymphocytes, 100 µL of recombinant protein (gp41 MN (565-771Delta642-725), HCV core protein, *Staphylococcus aureus* protein A), or diluted vehicle, with or without PLA2G1B, in 2.5% FBS RPMI was added to each well and the plates incubated for 2 h. Cells and supernatants were collected in Eppendorf tubes and centrifuged at 580 x g for 10 min at room temperature.

To study PLA2G1B activity on [3H]-AA-labeled CD4 T lymphocytes relative to that of human proPLA2G1B, cells were prepared as above but equilibrated in 2.5% FBS RPMI at 10^6^ CD4 T cells/400 µL/well in 24-well polystyrene plates for 90 min at 37°C in a humidified 5% CO2 atmosphere. Then, 100 µL medium or recombinant sPLA2 proteins in 2.5% FBS RPMI was added to each well and the plates incubated for 2 h.

The [3H]-AA released into the cell supernatant was quantified in 300 µL using 16 mL of Ultima gold (Perkin Elmer, 6013329) in low diffusion vials (Perkin Elmer, 6000477) on a scintillation counter (tri-Carb 2800 TR liquid scintillation analyzer, Perkin Elmer).

To evaluate the effect of the H48Q mutation, the results are expressed as PLA2G1B activity (release of [3H]-AA into the supernatant of cells treated with WT or H48Q PLA2G1B minus the spontaneous release of [3H]-AA by cells treated without PLA2G1B, in cpm/mL).

To evaluate the effect of the sPLA2 inhibitors, the results are expressed as the percentage of inhibition of PLA2G1B activity on cells treated with PLA2G1B and several doses of the inhibitors.

To evaluate the relative effect of PLA2G1B versus that of proPLA2G1B, the results are shown as the percentage of activity with proPLA2G1B relative to that of PLA2G1B activity.

To test the effect of viral or bacterial protein cofactors, the results are expressed as PLA2G1B activity (release of [3H] arachidonic acid into the supernatant of cells treated with recombinant proteins (gp41, HCV core or SA protein A) or buffer together with PLA2G1B minus the spontaneous release of [3H] arachidonic acid by cells treated with recombinant proteins alone or buffer only, without PLA2G1B, in cpm/mL).

### PLA2G1B Enzymatic Assay on [3H] Arachidonic Acid-Labeled Jurkat E6.1 T Cells

Jurkat E6.1 T cells (ECACC 88042803) or gC1qR KO Jurkat E6.1 T cells were incubated for 17 h at 5 x 10^5^ cells/mL with 1 µCi/mL arachidonic acid [5,6,8,9,11,14,15-^3^H(N)] (Perkin Elmer, NET298Z250UC) in RPMI 1640 medium (Lonza) supplemented with 10% FBS, 50 mM HEPES pH 7.4, glutamine, penicillin, streptomycin, and fungizone at 2 mL/well in 6-well plates at 37°C in a humidified 5% CO2 atmosphere. Cells were washed twice with RPMI with 10% FBS by centrifugation at 300 x g for 10 min at room temperature and then frozen in 90% FBS 10% DMSO at 10^7^ cells/mL/vial at -80°C. The percentage of [3H] arachidonic acid in Jurkat T cells (1 minus ratio of [3H] arachidonic acid in the Jurkat T-cell supernatant (cpm/mL) relative to the total [3H] arachidonic acid in the supernatant and cells (cpm/mL)) was measured to control the cell preparation.

To test PLA2G1B activity on [3H]-AA-labeled Jurkat E6.1 T lymphocytes, cells were thawed in 10% FBS RPMI preheated to 37°C, centrifuged at 300 x g for 10 min at room temperature, washed twice in 2.5% FBS RPMI, and equilibrated in 2.5% FBS RPMI at 5 x 10^4^ Jurkat E6.1 T cells/400µL/well in 24-well polystyrene plates for 90 min at 37°C in a humidified 5% CO2 atmosphere. Cells were pretreated with 50 µL of the peptides per well at 110 μM in 2.5% FBS RPMI for 2, 4, or 21 h, as indicated in the figures. Then, 50 µL of 2 µM PLA2G1B in 2.5% FBS RPMI or medium alone were added per well and the plates incubated for 2 h. Cells and supernatants were collected in Eppendorf tubes and centrifuged at 580 x g for 10 min at room temperature. The [3H] arachidonic acid released into the cell supernatant was quantified in 300 µL using 16 mL Ultima gold (Perkin Elmer, 6013329) in low diffusion vials (Perkin Elmer, 6000477) on a scintillation counter (tri-Carb 2800 TR liquid scintillation analyzer, Perkin Elmer).

Results are expressed as ΔPLA2G1B activity with the peptides minus PLA2G1B activity with the Scrambled 3S (the release of [3H] arachidonic acid into the supernatant of cells treated with PLA2G1B and peptide minus the release of [3H] arachidonic acid by cells treated with PLA2G1B and Scrambled 3S peptide, in cpm/mL).

### ELISA Study of PLA2G1B and gp41 Interaction

*Study of gp41 binding to coated PLA2G1B in ELISA.* Microplates were coated with 100 µL of PLA2G1B recombinant proteins at 10 µg/mL in protein-free blocking buffer (37572, Pierce). Then, the binding of serial dilutions (0-1 µg/well) of strep-tagged gp41 (D117 strain in A and MN strain in B) or unrelated proteins (CTL1: EFF-1 or CTL2: IF38 in A and CTL1: EFF1 in B) to PLA2G1B was revealed by the sequential addition of mouse anti-strep-tag mAb (MA5-17283, Invitrogen) and goat anti-mouse HRP-conjugated Ab (31430, Pierce), followed by the HRP reaction with TMB. All dilutions were tested in triplicate.

*Study of PLA2G1B binding to coated gp41 in ELISA.* Microplates were coated with 100 µL of gp41 (D117III strain in C and MN strain in D) recombinant proteins at 10 µg/mL in protein-free blocking buffer (37572, Pierce). Then, the binding of serial dilutions (0-1 µg/well) of PLA2G1B, to gp41 was revealed by the sequential addition of anti-PLA2G1B mAb (1C11, produced at BIOTEM, described in (1) and HRP-anti-mouse Ab, followed by the HRP reaction with TMB. All dilutions were tested in triplicate.

### Pull-Down Assay of PLA2G1B With gp41

Recombinant PLA2G1B (50 µg/mL) in PBS was incubated, or not, with recombinant gp41 (D117 III strains) with a strep-tag at 10 µg/mL in 1.5-mL Eppendorf tubes overnight (Test tube-rotor, 34528, Snijders, Netherlands) at 4°C. Then, gp41 was pulled-down by adding 50 µL of prewashed Strep-Tactin XT-beads (Mag strep “type 3” XT beads, IBA, 2-4090-002) per tube, followed by incubation for 30 min on ice with vortexing from time to time (4X). Beads were washed three times in washing buffer and the pulled-down protein complex was eluted in 50 µL 1X BTX buffer (IBA, 2-1042-025). Then, the proteins in 5 µL of flow through and 30 µL of the eluted sample were separated through 4%–20% Tris-Bis SDS-PAGE (BIO-RAD) gels under reducing conditions. Antigens were transferred to PVDF membranes (BIO-RAD) using a Trans-Blot Turbo (BIO-RAD). After blocking nonspecific binding sites with 5% milk/0.05% tween 20 in PBS, PLA2G1B was revealed using 10 µg/mL 1C11 mouse anti-PLA2G1B mAb and gp41 using 5 µg/mL polyclonal anti-gp41 (PA1-7219, Thermofisher). Goat anti-mouse (31430, Pierce) and donkey anti-goat (705-035-003, Jackson Immunoresearch) HRP-conjugated antibodies were used at a 1:10,000 dilution. Detection occurred directly on the membrane using SuperSignal West Pico Plus Substrate (34580, Thermofisher Scientific).

### Generation of gC1qR KO Jurkat E6.1 T Cells

The global strategy for the development of Jurkat cells deprived of C1QBP (the gene for gC1qR) was based on the design of a targeting vector permitting bi-allelic inactivation of the C1QBP gene *via* homologous recombination using CRISPR/Cas9 method as previously described in (10). Homologous human C1QBP regions isogenic with the Jurkat E6.1 T cell line (ECACC 88042803) were used. The targeting vector was synthesized by Genewiz and cloned into the pUC57-Amp vector. The third exon of the human C1QBP gene was targeted by introducing a neomycin resistance gene (NeoR) selection cassette, resulting in the interruption of the open reading frame of C1QBP. The NeoR cassette was cloned using BamHI/NotI restriction sites. The targeting vector was verified by DNA restriction digestion cut with selected restriction enzymes (APaL1, Drd1, Pvu1, Pvu2, BamH1/NotI, Not1/NcoI, NEB) and target region sequencing. The DNA primers corresponding to C1QBP sgRNA (1828-Crispr_1A: CACC-GAAGTGACCGTGATTCTAAAA and 1828-Crispr_1B: AAAC-TTTTAGAATCACGGTCACTTC) were hybridized and cloned (Quick Ligase - New England Biolabs, NEB) into the pX330 plasmid (Addgene, 42230; Feng Zhang, MIT) using the BbsI restriction site (NEB).

The Jurkat cells (5 x 10^6^) were resuspended in 100 μL of Opti-MEM and 7 μg of CRISPR/Cas9 plasmid and 2.5 μg of targeting vector were added. The cells were electroporated using a Nepa21 electroporator. After cell selection in G418 selective medium, the Jurkat cell clones were prescreened by PCR genotyping. Independent cell clones knocked-out for the C1QBP gene were amplified and verified by PCR genotyping and target region sequencing. Our validation pipeline for the independent Jurkat cell clones deficient for the C1QBP gene consisted of PCR genotyping. The genomic DNA of gene edited Jurkat cells was isolated by proteinase K treatment and phenol purification. Each cell clone with bi-allelic inactivation of the C1QBP gene was confirmed by PCR genotyping and by target region sequencing. PCR amplification was performed with Platinum HiFi Taq (Life technologies) for 2 min at 50°C with primers 1828_RH5_F: TACTACAGCCCTTGTTCTT and 1828_RH3_R: AGCACTTCCTGAAATGTT. The primers were designed to be in the C1QBP human locus and not in the homologous arms. The wild type (WT) and mutant alleles were distinguished in the same PCR reaction. The WT and mutant alleles resulted in 1,146-bp and 2,362-bp amplification products, respectively. This PCR genotyping protocol allowed the identification of the homozygous Jurkat cell clones knocked-out for both alleles of the C1QBP gene.

### Immunoblot Detection of gC1qR in Jurkat E6.1 T Cells

Western-blot analysis of gC1qR protein expression in WT and gC1qR KO Jurkat E6.1 T cell lysates was performed as follows. Cells were lysed in mammalian protein extraction reagent (M-PER, 11884111, Thermo Scientific) buffer and the amount of protein quantified using the BCA Protein assay kit on cleared supernatants (23227, Pierce, Thermo Scientific). An equal amount of total protein was loaded for WT and gC1qR KO Jurkat E6.1 T cells (40 µg) and fractionated by SDS-PAGE on 8-16% Mini-PROTEAN TGX Stain Free Gels (4568104, BIORAD), electro-transferred, and probed by immunoblotting using a specific antibody against gC1qR (60.11 Santa Cruz at 1:50, 74.5.2 Abcam at 1:1,000) or ß-actin (AC-74, Sigma at 1:2,000) in PBS-0.05% Tween-5% BSA at room temperature for 2 h and goat anti-mouse-IgG-HRP (1:20,000, 31430, Invitrogen) in PBS-0.05% Tween-5% BSA for 1 h. Bound antibodies were detected using the ECL immunoblotting detection system (NEL103001EA, PerkinElmer).

### Statistics

Statistical parameters, including the exact value of n, precise measures (mean ± SD in all Figures, with the exception of the mean ± SEM in **Figure 4**), statistical significance, and tests used for each analysis are reported in the figures and figure legends. Analyses were performed using GraphPad Prism (GraphPad Software Inc.).

For the pSTAT5-NT experiments, one donor represents one experiment. For the [3H]AA assays, the number of experiments is indicated and certain experiments were repeated on cells from the same donor. The number of donors is indicated in certain figure legends.

Correlations between two variables were evaluated by Pearson’s correlation and linear regression.

Data were analyzed using the two-tailed unpaired *t*-test for two groups or ANOVA with Tukey’s correction for multiple comparisons for data with a Gaussian distribution according to the D’Agostino & Pearson omnibus test. For data that were not Gaussian, the Mann-Whitney non-parametric test was used to compare two groups and the Kruskal-Wallis test when more than two groups were compared. If the Kruskal-Wallis test was significant, two-by-two comparisons were conducted to identify groups that differed, but applying a Bonferroni correction. The level of significance is indicated as *p < 0.05, **p < 0.01, and ***p < 0.001 in all figures.

## Results

### The Enzymatic Activity of PLA2G1B on CD4 T-Cell Membranes Is Increased by gp41

**Figure 1:**
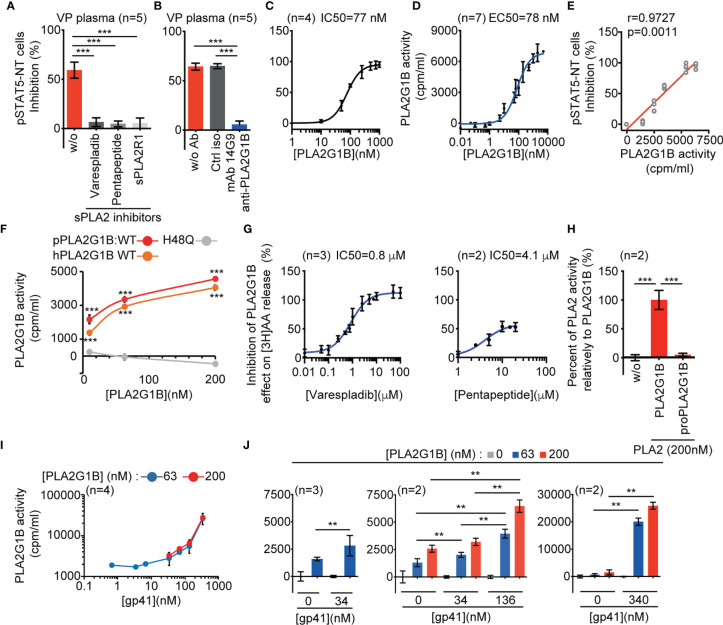
HIV gp41 protein increases PLA2G1B enzymatic activity on CD4 T-cell membranes. **(A, B)** The sPLA2 inhibitors varespladib, pentapeptide, and sPLA2R1 and the anti-PLA2G1B mAb 14G9 strongly inhibit PLA2G1B activity in HIV viremic plasma. Purified HD CD4 T cells from two donors **(A)** and three donors **(B)** were treated with 3% of VP plasma from five viremic HIV patients together with varespladib (50 µM), pentapeptide (15 µM), sPLA2R1 (160 nM), or not **(A)** or control isotype (ctrl iso) or 14G9 mAb (667 nM, B) and the pSTAT5-NT cell response to IL-7 was analyzed by confocal microscopy. **(C)** PLA2G1B inhibits the pSTAT5-NT cell response to IL-7 in a dose-response manner. HD CD4 T cells were purified from four donors. The IC50 value is indicated. Results are shown as the mean ± SD of the percentage of pSTAT5-NT cell inhibition for A-C. **(D)** PLA2G1B activity can be followed in a dose-response manner on human CD4 T cells labeled with tritiated arachidonic acid. CD4 T cells purified from four donors were incubated with several concentrations of PLA2G1B (0.1 nM-5 µM) for 2 h. Then, the release of [3H]-AA in the cell supernatant due to PLA2G1B activity was quantified with a radioactivity counter (tri-Carb 2800 TR liquid scintillation analyzer, Perkin Elmer). Results are shown as the mean ± SD of PLA2G1B activity in cpm/mL from a pool of seven experiments. The EC50 value is indicated. **(E)** pSTAT5-NT cell inhibition positively correlates with PLA2G1B enzymatic activity on [3H]-AA-labeled CD4 T cells. Potential correlations were analyzed using the Pearson r test and a linear regression is presented. **(F)** [3H]-AA release is dependent on PLA2G1B enzymatic activity. [3H]-AA-labeled CD4 T cells were treated with several concentrations (10, 63, 200 nM) of WT human (hPLA2G1B) or WT or the catalytic-site mutant H48Q porcine PLA2G1B (pPLA2G1B). Results are shown as the mean of PLA2G1B activity ± SD of one experiment with the treatment in triplicate. **(G)** Varespladib, and pentapeptide strongly inhibit PLA2G1B enzymatic activity on [3H]-AA-labeled CD4 T cells. Results are shown as the percentage of inhibition of PLA2G1B activity on cells treated with PLA2G1B (65 nM) and several doses of inhibitors. IC50 values are presented. **(H)** ProPLA2G1B is inactive in the [3H]-AA release assay on CD4 T-cell membranes. [3H]-AA-labeled CD4 T cells were treated in triplicate with PLA2G1B or proPLA2G1B at 200 nM. Results are shown as the percentage of activity with proPLA2G1B relative to that of PLA2G1B. **(I, J)** HIV gp41 increases PLA2G1B activity in a dose-dependent manner on human CD4 T cells. [3H]-AA-labeled CD4 T cells were incubated alone or with PLA2G1B (63 nM or 200 nM) in the presence or not of several concentrations of recombinant gp41 protein (0.68 nM-340 nM). Results are shown as the mean ± SD from a pool of four experiments for I and n = 2-3 experiments for **(J)** **p < 0.01 and ***p < 0.001 by two-way ANOVA with Tukey’s correction for multiple comparisons **(A, B, F, H)** and the Kruskal-Wallis test, followed by the Mann-Whitney test with p-values adjusted for multiple comparisons between groups **(J)**. For F, only comparisons between H48Q and WT hPLA2G1B or pPLA2G1B at each PLA2G1B concentration are shown.

To formally demonstrate that the effect of HIV VP plasma on CD4 T cells is due to the enzymatic activity of PLA2G1B we performed additional experiments to complete our previous article (1). We first determined the IC50 of three different PLA2 inhibitors, varespladib, pentapeptide, and soluble mouse PLA2R1 receptor (sPLA2R1), on PLA2G1B protein in our standard IL-7-induced pSTAT5-NT inhibition assay (**Supplementary Figure 1A**). Based on the determined IC50s, we treated VP plasma with the inhibitors. All three almost completely blocked the effect of VP plasma on CD4 T cells (**Figure 1A**). We obtained similar results when VP plasma was treated with the anti-PLA2G1B mAb 14G9, selected to inhibit the enzymatic activity of PLA2G1B (**Figure 1B**). These results confirm that the inhibitory activity of VP on the CD4 T-cell response is dependent on the enzymatic activity of PLA2G1B.

We previously showed that gp41 is involved in the inhibitory activity of PLA2G1B in VP plasma and that it can increase such activity on the pSTAT5-NT response in CD4 T cells (1). This effect is likely due to an increased enzymatic activity of PLA2G1B on the CD4 T-cell membrane. Thus, we developed an assay to directly study the effect of gp41 on the enzymatic activity of PLA2G1B on the cell surface membranes of CD4 T cells. PLA2 family proteins are known to release fatty acids by digesting lipid membranes. One of the hallmarks of PLA2G1B activity on cell membranes is the release of arachidonic acid (AA). Thus, we labeled primary CD4 T cells purified from HD with tritiated arachidonic acid [3H]AA. The IC50 of the inhibitory activity of PLA2G1B on the pSTAT5-NT response in CD4 T cells (IC50 = 77 nM, **Figure 1C**) was very similar to the EC50 of PLA2G1B activity measured by monitoring the release of [3H]AA in cell supernatants (EC50 = 78 nM, **Figure 1D**), with a release of AA proportional to the PLA2G1B concentration. More remarkably, there was a positive correlation between the release of AA and the inhibition of pSTAT5-NT (**Figure 1E**). To demonstrate that the [3H]AA release upon PLA2G1B treatment of CD4 T cells is an appropriate assay to study the enzymatic activity of PLA2G1B, we studied the effect of the catalytic-site mutant H48Q (**Figure 1F**) and that of several PLA2 enzymatic inhibitors (**Figure 1G** and **Supplementary Figure 1B**) in this assay. H48Q was previously shown to inhibit the PLA2G1B-dependent induction of aMMDs, inhibition of pSTAT5-NT and digestion of phosphatidylserine on the membrane of primary human CD4 T cells (1). As expected, the catalytic-site mutant H48Q showed no activity in this assay (**Figure 1F**) and all PLA2 enzymatic inhibitors, varespladib, pentapeptide (**Figure 1G**), sPLA2R1, and the anti-PLA2G1B mAb 14G9 (**Supplementary Figure 1B**), inhibited the [3H]AA release in a dose-dependent manner. Furthermore, inactive proPLA2G1B, in which the propeptide blocks the active catalytic site and enzymatic activity, did not induce [3H]AA release (**Figure 1H**). In further support of the correlation between pSTAT5-NT inhibition and PLA2G1B enzymatic activity (**Figure 1E**), the IC50s of varespladib, pentapeptide and sPLA2R1 in the [3H]AA release assay (**Figure 1G** and **Supplementary Figure 1B**) were similar to those in the pSTAT5-NT assay (**Supplementary Figure 1A**). In addition, the enzymatic activity showed complete specificity relative to PLA2GIIA and PLA2GIID but PLA2GX was more active than PLA2G1B (**Supplementary Figure 1C**). Notably, the inhibitory effect in HIV VP plasma was blocked by anti-PLA2G1B but not anti-PLA2GX Abs (1) and PLA2GX protein was not detected in HIV plasma. Overall, these results suggest that the enzymatic activity of PLA2G1B in HIV VP plasma on CD4 T-cell membranes controls intracellular events, such as pSTAT5-NT.

We observed that recombinant gp41 protein increases PLA2G1B enzymatic activity on the membrane of CD4 T cells in a gp41 dose-dependent manner using the [3H]AA release assay (**Figure 1I**), with a significant increase from 34 to 340 nM of gp41 (**Figure 1J**, *p* < 0.01). The observation that the activity of PLA2G1B was significantly higher with 136 nM than 34 nM of gp41 for each PLA2G1B concentration tested (middle panel, **Figure 1J**, *p* < 0.01) confirms that PLA2G1B activity is regulated by gp41 in a dose-dependent manner. PLA2G1B activity was more than 20-fold higher with 340 nM of gp41 than with PLA2G1B alone (63 nM, *p* < 0.0022). Overall, these results confirmed our previous observations using the pSTAT5-NT assay. Gp41 can increase the effect of PLA2G1B on CD4 T cells and showed that gp41 directly increases the enzymatic activity of PLA2G1B on the membranes of CD4 T cells.

### PLA2G1B Directly Interacts With gp41 Protein

**Figure 2:**
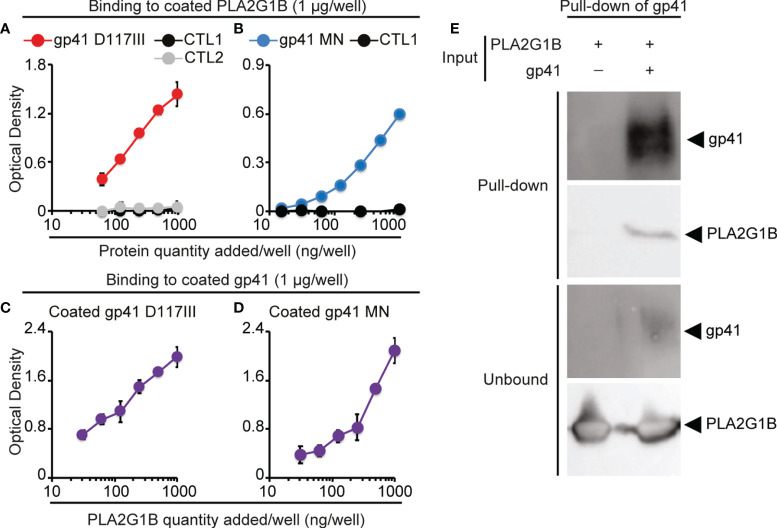
PLA2G1B directly binds to gp41. **(A, B)** gp41 binds to coated PLA2G1B by ELISA. Binding to coated PLA2G1B of serial dilutions (0-1 µg/well) of gp41 (D117III strain on A and MN strain on B), or unrelated proteins (CTL1: EFF-1 on A and B or CTL2: IF38 on A). **(C, D)** PLA2G1B binds to coated gp41 by ELISA. Binding of serial dilutions (0-1 µg/well) of PLA2G1B to coated gp41 (D117III on C, or MN on D) was tested in triplicate by ELISA. Results are shown as the mean ± SD of the OD value of one representative experiment among two **(A)**, five **(B)**, one **(C)** and five **(D)**. **(E)** gp41 binds to PLA2G1B in pull-down assays. Recombinant PLA2G1B protein was incubated with strep-tagged-gp41 (D117III strain, gp41+PLA2G1B) or not (PLA2G1B only). PLA2G1B-gp41 complexes were pulled-down with strep-tactin XT-beads that pull-down gp41. Pulled-down products and unbound proteins were revealed by immunoblotting with goat polyclonal anti-gp41 Ab or mouse anti-PLA2G1B mAb. One representative experiment of five experiments with similar results with D117III gp41 is presented. Similar results were obtained in three experiments with MN gp41 (**Supplementary Figure 2**).

Gp41 has been shown to bind to CD4 T-cell membranes (4). It led us to postulate that gp41 increases the enzymatic activity of PLA2G1B on CD4 T cells by interacting with CD4 T-cell membranes and PLA2G1B, which would increase the PLA2G1B concentration at the cell membrane and, thus, its enzymatic activity. We first tested the interaction of gp41 and PLA2G1B by solid-phase microplate binding assays. Gp41 from two different HIV strains, D117III and MN, bound to PLA2G1B when it was used to coat microwells, whereas other control proteins with the same tag, to reveal protein binding, did not (CTL1: EFF1 (11), CTL2: IF38 (12), **Figures 2A, B**). The reverse experiments with coated gp41, from the same two HIV strains (**Figures 2C, D** *)* confirmed the interaction between PLA2G1B and gp41. We then tested the interaction of recombinant gp41 and PLA2G1B in solution *in vitro* by pull-down assays of gp41. When gp41 was pulled-down (**Figure 2E**) we observed that PLA2G1B was present in the pulled-down proteins (**Figure 2E**), whereas no PLA2G1B was observed upon pull-down with beads in the absence of gp41 (**Figure 2E**). This experiment was repeated five times with gp41 D117III and three times with gp41 MN (**Supplementary Figure 2**). Therefore, these results indicate that PLA2G1B can directly bind to gp41. It suggests that this interaction could increase PLA2G1B activity on CD4 T-cell membranes after the binding of gp41 to the membrane.

### Several gC1qR-binding Proteins Encoded by Human Pathogens can Increase PLA2G1B Activity on CD4 T-Cell Membranes

Gp41 and its conserved sequence 3S are known to bind to the gC1qR (4, 5). We previously showed that inhibition of the CD4 T-cell response due to PLA2G1B activity in VP plasma is 3S-dependent and that the 3S peptide alone can increase the inhibitory activity of PLA2G1B on CD4 T cells (1). Thus, we postulated that gp41 may bind to the gC1qR to increase PLA2G1B activity on CD4 T-cell membranes. The gC1qR is an innate sensor known to bind to several proteins of microbial origins (6). One possibility is that the gC1qR-dependent regulation of PLA2G1B activity may be a common mechanism used by pathogens to inhibit CD4 T-cell responses and impair the immune response.

**Figure 3:**
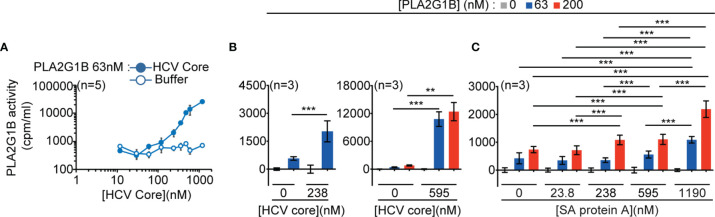
PLA2G1B activity on CD4 T cells is increased by several gC1qR-binding microbial proteins. **(A, B)** HCV core increases PLA2G1B activity on human CD4 T cells in a dose-dependent manner. [3H]-AA-labeled CD4 T cells were incubated alone or with PLA2G1B (63 nM or 200 nM) in the presence or not of several concentrations of recombinant HCV Core protein (11.9 nM-1190 nM) or equivalent concentrations of buffer alone (Buffer for A or 0 nM for B). **(C)** *Staphylococcus aureus* (SA) protein A increases PLA2G1B activity on human CD4 T cells in a dose-dependent manner. [3H]-AA-labeled CD4 T cells were incubated alone or with PLA2G1B (63 nM or 200 nM) in presence or not of several concentrations of SA protein A (23.8 nM-1190 nM). Results are shown as the mean ± SD from a pool of five experiments for A, and three experiments for B and C. **p < 0.01 and ***p < 0.001 by the Kruskal-Wallis test followed by the Mann-Whitney test, with p-values adjusted for multiple comparisons between groups.

We thus tested the effect of two representative microbial proteins known to bind to the gC1qR (6) on the enzymatic activity of PLA2G1B on CD4 T-cell membranes: the viral HCV core protein (5, 13) and the bacterial protein A of *Staphylococcus aureus* (SA) (14, 15). Both HCV core protein (**Figures 3A, B**) and SA protein A (**Figure 3C**) significantly increased the enzymatic activity of PLA2G1B on the membranes of CD4 T cells in a microbial protein dose-dependent manner. HCV core protein was a very potent inducer of PLA2G1B enzymatic activity. The effect of HCV core was significant with 238 nM of HCV core (**Figure 3B**, *p* < 0.001). PLA2G1B (at 63 nM) activity was 26-fold higher in the presence of 595 nM HCV core than PLA2G1B alone at the same concentration (**Figure 3B**). Although SA protein A was also a significant inducer of PLA2G1B enzymatic activity (**Figure 3C**, *p* < 0.001), the effect was less strong, showing only three-fold more PLA2G1B activity with 1,190 nM SA protein A and PLA2G1B (at 200 nM) than with PLA2G1B alone. Because gp41, HCV core, and SA protein A are all gC1qR-binding proteins, these results support the hypothesis that several microbial proteins could play the role of cofactor in the PLA2G1B/cofactor system and increase the enzymatic activity of PLA2G1B *via* the binding to the gC1qR.

### gC1qR Plays a Critical Role in the Regulation of PLA2G1B Enzymatic Activity by gp41

**Figure 4:**
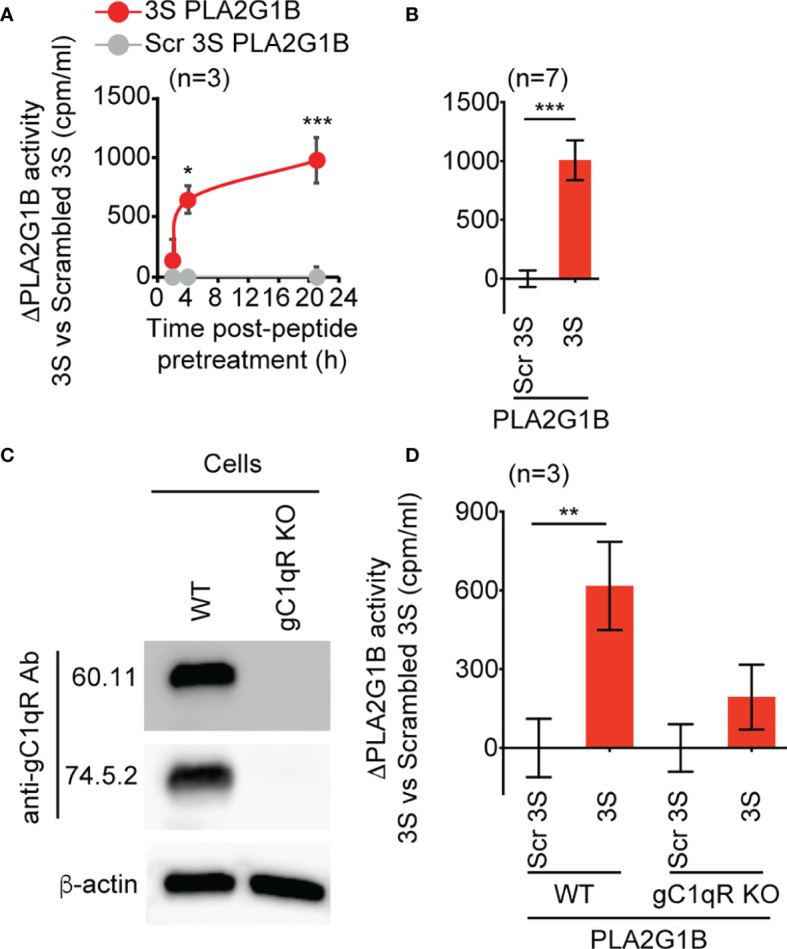
The 3S/gp41 peptide regulates PLA2G1B activity in a gC1qR-dependent manner. **(A, B)** The HIV 3S peptide increases PLA2G1B activity on human Jurkat T cells. [3H]AA-labeled Jurkat T cells were pretreated with 3S gp41 (3S) or scrambled 3S (Scr 3S) peptides (11 µM) for various times (2, 4, or 21 h for A) or 21 h (for B) and incubated alone or with PLA2G1B (200 nM). **(C)** The gC1qR protein is detected in WT but not in gC1qR-deficient (gC1qR KO) Jurkat T cells by immunoblot. Two different anti-gC1qR mAbs were used (60.11 and 74.5.2), as well as anti-ß-actin mAb as an endogenous control of protein load. **(D)** The 3S peptide increases PLA2G1B activity on WT but not on gC1qR KO cells. WT and gC1qR KO [3H]-AA-labeled Jurkat T cells were pretreated with 3S or Scr 3S peptides at 11 μM for 21 h. Then, peptide-pretreated cells were incubated with PLA2G1B (200 nM) for 2 h. Results are expressed as the mean ± SEM of a pool of three **(A, D)** and seven **(B)** experiments performed in triplicate and as ΔPLA2G1B activity (activity with the 3S peptide minus that with the Scr 3S peptide). The level of [3H]AA released in the cell supernatant was quantified in cpm/mL. *p < 0.05, **p < 0.01 and ***p < 0.001 by two-way ANOVA with Tukey’s correction for multiple comparisons **(A, D)** or an unpaired t-test **(B)**.

We sought a CD4 T-cell line that could be labeled with [3H]AA to directly demonstrate the role of the gC1qR in the regulation of PLA2G1B activity by microbial proteins. We used the 3S peptide, as it has been shown to bind to the gC1qR (4, 5). We found that gC1qR is expressed by Jurkat T cells and that the gp41-derived 3S peptide (**Figures 4A, B**) can increase the enzymatic activity of PLA2G1B on the membranes of Jurkat T cells. The effect of the 3S peptide required pretreatment of the Jurkat T cells for 4 or 21 h before the addition of PLA2G1B to significantly increase its enzymatic activity relative to scrambled 3S peptide-treated cells, with a major effect occurring after 21 h of pretreatment (**Figure 4A**, *p* < 0.0001). We confirmed this last experimental condition to be the best for studying the effect of 3S on the enzymatic activity of PLA2G1B in several experiments (**Figure 4B**, *p* < 0.0001).

We then generated clones of Jurkat T cells deprived of gC1qR (gC1qR KO) using the CRISPR-Cas9 method. We confirmed the loss of the gC1qR protein in gC1qR KO cells relative to WT Jurkat CD4 T cells by immunoblotting using two anti-gC1qR mAbs that bind to different epitopes of the gC1qR protein (**Figure 4C**). Notably, 3S peptide significantly increased the enzymatic activity of PLA2G1B on WT cells relative to scrambled 3S peptide (*p* < 0.01), whereas there was no significant difference in PLA2G1B activity between 3S and scrambled 3S peptide-treated cells deficient for the gC1qR (**Figure 4D**). Overall, these results show that gp41 can increase PLA2G1B activity through its conserved 3S region in a gC1qR-dependent manner.

### 3S-Like Motifs Are Present in Proteins Encoded by Several Human Pathogens

We next screened protein sequence databases for similarity to identify other microbial proteins that could regulate the enzymatic activity of PLA2G1B on CD4 T cells and inhibit the immune response using Blastp with an expect threshold of 100 and a 3S amino-acid substitution matrix (AASM) based on the 3S sequence of the peptide with cofactor activity (**Figure 4** and reference (1) and major amino-acid substitutions found in natural HIV-1 sequence variants (**Supplementary Figure 3**). We identified 42 peptides with 3S-like motifs (**Supplementary Figure 3**). Eleven selected peptides encoded by human pathogens are presented in **Supplementary Table 1**. They are derived from eight bacterial species (*Porphyromonas gingivalis* (7, 8, 16, 17), *Proteus mirabilis* (18), *Leptospira weilii* (19), *Terrisporobacter glycolicus* (20), *Bacteroides fragilis* (21, 22), *Aggregatibacter actinomycetemcomitans* (23, 24), *Porphyromonas somerae* (25), *Aggregatibacter aphrophilus* (23, 26) and one fungus (*Candida glabrata* (27, 28) that are involved in several human infections or have been shown to be associated with autoimmune diseases or with an increased risk of developing cancer (more detailed references on these pathogens are provided in **Supplementary Table 1** and in the **supplementary Material File**). These results show that 3S-like motifs are not restricted to HIV or viruses but are also present in several pathogens, such as bacteria and fungi.

### PLA2G1B Plays a Role in the Inhibitory Activity of Pancreatic Ductal Adenocarcinoma Plasma on the pSTAT5-NT Response in CD4 T Cells

Among pathogens encoding proteins with 3S-like motifs, bacterial infection with *P. gingivalis* has been shown to be associated with an increased risk of gastrointestinal cancer, including pancreatic cancer (7–9). This pathogen encodes seven peptides with 3S-like motifs (**Supplementary Figure 3**). Based on previous publications, amino acids W6, S7, N8, and S10 of the 3S HIV peptide PWNAS**WSN**K**S**LDDIW (**Supplementary Table 1**) are the most critical for induction of the NKp44L by the 3S peptide (29). This effect is due to the binding of 3S to the gC1qR (4), as for the increased of PLA2G1B activity described here. We thus focused on the OmpA pg peptide of *P. gingivalis*, which is the only one from this pathogen that contains W6, S7, N8, and S10 (**Supplementary Table 1** and **Supplementary Figure 3**). We postulated that the OmpA Pg peptide may regulate PLA2G1B activity. If this is true, it is possible that PDAC plasma may contain PLA2G1B activity that inhibits the CD4 T-cell response, based on our previous findings with HIV VP plasma. Thus, 3S-like peptides derived from *P. gingivalis* could play the role of cofactor in increasing PLA2G1B activity.

**Figure 5:**
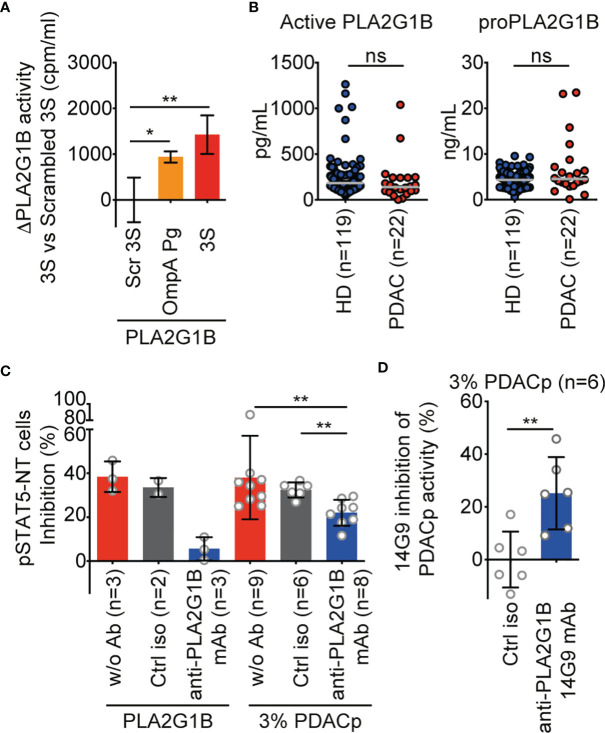
PLA2G1B is involved in PDAC plasma inhibition of the CD4 T-cell pSTAT5-NT response. **(A)** A 3S-like peptide from *P. gingivalis* (OmpA Pg) increases PLA2G1B enzymatic activity. [3H]-AA-labeled Jurkat T cells were pretreated with 3S, OmpA Pg or scrambled 3S (Scr 3S) peptides (11µM) for 21 h and incubated alone or with PLA2G1B (200 nM). Results are shown as the mean ± SD of PLA2G1B activity with the 3S or OmpA Pg minus PLA2G1B activity with Scr 3S from one representative experiment of two with similar results. **(B)** ELISA quantification of active PLA2G1B and proPLA2G1B in plasma from HD and PDAC donors (the median is shown). **(C)** Anti-PLA2G1B mAb inhibits PLA2G1B activity in PDAC plasma. HD CD4 T cells from three donors were treated with PLA2G1B (75 nM), 3% of PDAC plasma (PDACp) alone (w/o Ab, n = 9 plasma samples), with control isotype (ctrl iso, 667 nM, n = 6 plasma samples) or anti-PLA2G1B mAb (14G9, 667 nM, n = 8 plasma samples) and the pSTAT5-NT cell response to IL-7 was analyzed by confocal microscopy. Results are shown as the mean ± SD of percentage of pSTAT5-NT cells inhibition. **(D)** Heterogeneity of anti-PLA2G1B mAb 14G9 inhibition of PLA2G1B activity in PDAC plasma. Results are shown as the mean ± SD of the percentage of inhibition of PDACp activity from six patients on pSTAT5-NT by 14G9 relative to that of control isotype-treated plasma. *p < 0.05, and **p < 0.01, by two-way ANOVA with Tukey’s correction for multiple comparisons for A, by the Mann-Whitney test for B and D, and by the Kruskal-Wallis test followed by the Mann-Whitney test, with p-values adjusted for multiple comparisons between groups, for C. ns, non significant.

We first tested the effect of the OmpA Pg peptide on the enzymatic activity of PLA2G1B in parallel with the 3S peptide, used as a positive control (**Figure 5A**). As for 3S from gp41, OmpA Pg increased PLA2G1B activity over that of cells treated with scrambled 3S. We also found there to be no difference between the active PLA2G1B and proPLA2G1B concentrations in PDAC and HD plasma (**Figure 5B**), similar to our observations for HIV VP and HD plasma (1).

Treatment of CD4 T cells with 3% PDAC plasma (PDACp) impaired the pSTAT5-NT response to IL-7 (38% inhibition) to a similar level as recombinant PLA2G1B protein (38% of inhibition, **Figure 5C**). Furthermore, this effect was partially dependent on PLA2G1B activity, as anti-PLA2G1B mAb significantly inhibited the activity of PDACp relative to cells treated with plasma alone or plasma treated with a control isotype antibody (*p* < 0.01, **Figure 5C**). Anti-PLA2G1B mAb (14G9) significantly inhibited PDACp activity relative to a control isotype (*p* < 0.01, **Figure 5D**). PLA2G1B appeared to be involved in the plasma of only certain PDAC patients, with 25 to 45% inhibition of PDAC plasma activity by anti-PLA2G1B mAb for 66% (4/6) of the plasma samples tested (**Figure 5D**). Overall, these data suggest that plasma from patients with PDAC can inhibit the CD4 T-cell response to IL-7 by a mechanism likely similar to that we describe for HIV plasma involving the PLA2G1B/cofactor system. It is possible that the PLA2G1B activity in PDAC plasma may be increased by a cofactor derived from *P. gingivalis*, as it encodes a protein containing a 3S-like motif that increases PLA2G1B enzymatic activity at the cell surface membrane.

## Discussion

In a recent study, we demonstrated that the PLA2G1B/cofactor system is an inhibitor of CD4 T cells that is involved in the induction of anergy and lymphopenia of CD4 T cells in HIV-infected patients (1). PLA2G1B induces aMMDs on CD4 T cells that trap and inactivate cytokine receptors. PLA2G1B inhibitory activity in HIV VP plasma is dependent on the HIV-1 gp41 envelope protein. We showed that gp41, through a conserved 3S motif, drives PLA2G1B activity on CD4 T cells. However, the mode of action of gp41 to increase PLA2G1B activity on CD4 T cells was yet to be elucidated.

We investigated this mechanism by developing a radioactive assay based on the measurement of [3H]AA release upon treatment of [3H]AA-labeled CD4 T cells with PLA2G1B. We demonstrated that this assay measures the enzymatic activity of PLA2G1B, as the H48Q catalytic site mutant was inactive (**Figure 1F**) and all PLA2G1B enzymatic inhibitors studied (varespladib, pentapeptide, soluble mouse PLA2R1 receptor), a neutralizing anti-PLA2G1B mAb, and the propeptide inhibited the release of [3H]AA by PLA2G1B (**Figures 1G, H** and **Supplementary Figure 1B**). Moreover, PLA2G1B enzymatic activity measured by this assay (**Figure 1D**) is strongly and positively correlated with PLA2G1B inhibitory activity on CD4 T cells as measured by the inhibition of IL-7-induced pSTAT5-NT (**Figures 1C, E**). More importantly, we showed that gp41 increases the enzymatic activity of PLA2G1B on the membranes of CD4 T cells (**Figures 1I, J**), explaining the gp41-driven increase of the inhibitory activity of PLA2G1B on CD4 T-cell response to IL-7 that we previously observed with the plasma of HIV-infected viremic patients. We also further defined the various steps of the mechanism involved.

*In vitro*, high amounts of PLA2G1B (from 10 nM to 200 nM) directly acted on CD4 T-cell membranes (**Figure 1D**). This indicates that its substrates (phospholipids) are spontaneously expressed at the surface of CD4 T cells. However, the concentration of PLA2G1B in the plasma of VP is very low (0.025 nM) and thus it cannot act alone. Therefore, cofactor(s) act at the surface of CD4 T cells by either increasing the presence of PLA2G1B or by modifying the quality or quantity of substrate accessible to PLA2G1B. Two types of mechanism are possible.

Here, we showed that gp41 directly interacts with PLA2G1B by solid-phase microplate binding and pull-down assays (**Figure 2**). Gp41 was shown to bind *via* its conserved 3S domain to the gC1qR on CD4 T-cell membranes (4). In addition, the 3S peptide increased PLA2G1B enzymatic activity on WT but not on gC1qR KO Jurkat cells (**Figure 4**). Overall, these results suggest that a gp41-PLA2G1B complex may bind to the gC1qR, resulting in an increase in the PLA2G1B concentration at the CD4 T-cell membrane followed by an increased enzymatic activity on the membrane and the inhibition of CD4 T-cell function.

Alternatively, another mechanism concerning the mode of action of the PLA2G1B/gp41 pair is possible. Based on the pluripotent role of the gC1qR (6), gp41 could also act by changing the membrane composition of CD4 T cells by a gC1qR-dependent mechanism that would increase the concentration of the phospholipid substrates of PLA2G1B, thus increasing its activity. Consistent with this hypothesis, it was shown that 3S binding to gC1qR can induce the fusion of exocytotic vesicles with CD4 T-cell membranes (4). The fusion of exocytotic vesicles with the plasma membrane changes its lipid composition, which could also increase PLA2G1B activity. Both mechanisms, together or independently, could increase the fluidity of the membrane and the formation of aMMDs, which induces anergy of the CD4 T cells by blocking the function of physiological receptors.

The gC1qR is an innate sensor that binds to several pathogens (6). Our discovery of a gC1qR-dependent activation of PLA2G1B by gp41 led us to hypothesize that other gC1qR-binding microbial proteins could also increase PLA2G1B activity. We demonstrate that two other microbial proteins that bind to gC1qR, HCV core and *Staphylococcus aureus* protein A, also increase the enzymatic activity of PLA2G1B on CD4 T cells (**Figure 3**). These observations suggest that PLA2G1B may also be involved in hepatitis C virus infection or several diseases due to *Staphylococcus aureus* infection. HCV core has been shown to bind to the gC1qR and to inhibit T-cell responsiveness and has been suggested to play a role in the persistence of HCV (13). Thus, PLA2G1B activity could also be increased by HCV core binding to the gC1qR in HCV-infected patients and contribute to the inhibition of T-cell responses and HCV persistence. *Staphylococcus aureus* infections, such as superficial skin infections (30), infective endocarditis (31) and sepsis (32) could also be affected by the PLA2G1B/SA protein A cofactor pair.

Importantly, we found that 3S-like motifs are present in several human infectious pathogens (**Supplementary Table 1** and **Supplementary Figure 3**) and that the one we tested increases the enzymatic activity of PLA2G1B (**Figure 5A**). This peptide is encoded by *P. gingivalis*, a Gram-negative anaerobic bacteria involved in periodontitis infection that is also associated with an increased risk of gastrointestinal cancer, including PDAC (90% of the pancreatic cancers) (7–9, 16). Among oral cancers, *P. gingivalis* has been found in oral squamous cell carcinoma (OSCC) and its role in carcinogenesis has been directly established (33). Numerous studies have shown that *P. gingivalis* may have systemic effects through its LPS (34). Very recently, *P. gingivalis* was directly identified inside pancreatic tumors (7). PDAC has also been shown to be associated with immune dysfunctions involving a paucity of dendritic cells (35) and the upregulation of PD-L1 (36). T-cell dysfunction, as well as decreased CD4 and CD8 counts, have also been described in PDAC (37), as in the terminal stages of HIV-infected patients.

The results reported here may partially explain such immunological defects. Plasma from PDAC patients diminished IL-7-induced pSTAT5-NT of HD CD4 T cells (**Figure 5C**). This inhibitory activity was partially due to PLA2G1B, as anti-PLA2G1B mAb, but not a control isotype, partially blocked the effect (**Figure 5C**). A detailed analysis of the effect of an anti-PLA2G1B mAb relative to that of a control isotype antibody on six plasma samples showed heterogeneous involvement of PLA2G1B. Only 25 to 45% specific inhibition of PDAC plasma activity with anti-PLA2G1B mAb was found in the plasma of 4/6 patients (**Figure 5D**). Heterogeneity of this response may reflect the stage of the disease, as well as the intensity of the degradation of the immune system. Further studies should be performed to clarify this point. One of the hallmarks of PLA2G1B/cofactor activity on CD4 T cells is the release of arachidonic acid. Notably, high levels of arachidonic acid in plasma, which could be due to higher PLA2G1B/cofactor system activity, have been positively associated with the risk of pancreatic cancer (38).

At both the cellular and molecular level, our data support the hypothesis that the PLA2G1B/cofactor system may be involved at least in certain patients. The hypothesis that *P. gingivalis* may be a cofactor merits further investigation. The 3S-like motif is exposed at the surface of the bacteria, as it is part of an outer membrane protein called OmpA family protein (WP_097552551.1 in the NCBI protein database, **Supplementary Figure 3** and **Supplementary Table 1**). In addition, it may act directly on CD4 T cells (39) and on their membranes, as OmpA Pg peptide increases PLA2G1B enzymatic activity (**Figure 5A**). The role of the PLA2G1B/cofactor system in PDAC plasma needs to be further investigated. Such studies would help our understanding of the immune disorders found in PDAC patients.

The origin of the two components of the PLA2G1B/cofactor system need to be clarified. We previously found that the major source of PLA2G1B is the pancreas, followed by the duodenum, jejunum, and ileum [see **Supplemental Data** in (1)]. We also observed that *pla2g1b* transcripts are primarily expressed in the pancreas, followed by the duodenum, whereas they were found in low amounts in lymphoid cells, including CD4 T cells, and they were almost undetectable in myeloid cells (see **Supplemental Data** in [1)]. According to these results, active PLA2G1B is mainly produced in the pancreas and intestinal tract from an inactive precursor (proPLA2G1B) that is activated by trypsin digestion and then participates in the digestion of phospholipids. The leaking of PLA2G1B may explain the presence of proPLA2G1B and active PLA2G1B in the blood. The origin of the cofactor appears to be specific for each disease. Taking the examples studied here, it may be a component of an external protein of the microorganism (HIV, *P. gingivalis* and *Staphylococcus aureus*) or an internal protein recovered in the plasma after degradation of the microorganism. These cofactors are proteins with 3S-like motifs and proteins that bind to gC1qR and drive PLA2G1B activity *via* the gC1qR. As PLA2G1B is the active component of the PLA2G1B/cofactor system, the therapeutic potential of anti-PLA2G1B mAbs should also be tested in certain pathologies that involve the PLA2G1B/cofactor system to determine whether it will be a useful candidate to block PLA2G1B activity and restore CD4 T cells to improve immune responses and patient outcomes.

In conclusion, we provide lines of evidence of a PLA2G1B/cofactor system that inhibits CD4 T-cell function. It helps understanding of the physiopathology of various infections, including some infections involved in oncogenesis. This study further demonstrates the capacity of microbes to disable the immune system by hijacking the activity of a natural endogenous enzyme and making it harmful. Our results establish this study as a pivotal element in the understanding of impaired CD4 T-cell immune response upon infection.

## Data Availability Statement

The raw data supporting the conclusions of this article will be made available by the authors, without undue reservation.

## Ethics Statement

The studies involving human participants were reviewed and approved by “Comité Consultatif de Protection des Personnes dans la Recherche Médicale” under the number 05-15 and was supported by the ANRS for the study of the effect of VP plasma on the pSTAT5-NT response of CD4 T cells. The study of PDAC plasma was registered under the number DC-2021-4516 by the Ministère de l’Enseignement Supérieur, de la Recherche et de l’Innovation. The patients/participants provided their written informed consent to participate in this study.

## Author Contributions

JP and JT conceived and designed the study. JP, AM, FB, and LJ conducted experiments at DIACCURATE. JP analyzed all data and performed enzymatic activity assays and LT provided expertise and feedback in the setting up of the assay. AM performed gp41 and PLA2G1B interaction studies. FB performed pSTAT5-NT assays. LJ performed ELISAs. AGS and BG provided expertise and feedback in the design of experiments to test the role of gC1qR. FF generated gC1qR KO cells. JP, JT and PP were involved in the identification of 3S-like motifs in microbes. AS selected and collected plasma samples from PDAC patients. JP and JT supervised the study. JT and PP secured funding. JP and JT wrote the manuscript with input from all authors. All authors contributed to the article and approved the submitted version.

## Funding

This work was part of the ANRS program EP33.

## Conflict of Interest

JT is cofounder and CSO of DIACCURATE, a spin-off of the Institut Pasteur. JP, AM, FB, and LJ are, or were, employees of DIACCURATE. PP was employed by company Truffle Capital. BG receives royalties from the sale of anti-gC1qR antibodies and gC1qR detection assay kit.

The remaining authors declare that the research was conducted in the absence of any commercial or financial relationships that could be construed as a potential conflict of interest.

## Publisher’s Note

All claims expressed in this article are solely those of the authors and do not necessarily represent those of their affiliated organizations, or those of the publisher, the editors and the reviewers. Any product that may be evaluated in this article, or claim that may be made by its manufacturer, is not guaranteed or endorsed by the publisher.
