# Supplementary material for: Microbial Protein Binding to gC1qR Drives PLA2G1B-Induced CD4 T-Cell Anergy
Source: Front Immunol. 2022 Mar 22;13:824746. doi: 10.3389/fimmu.2022.824746 (PMC8981723; doi:10.3389/fimmu.2022.824746)
Supplement: Supplementary file 1 [file DataSheet_1.pdf]

Supplementary Material

Supplementary Data

Supplementary Materials and Methods

Effect of sPLA2 inhibitors on PLA2G1B activity on the phosphorylation and nuclear translocation of STAT5 (pSTAT5-NT).

STAT5 phosphorylation and nuclear translocation in HD CD4 T cells were analyzed by microscopy after IL-7 stimulation (2 nM), or in HD CD4 T cells incubated with human PLA2G1B recombinant proteins (30 min), with or without a pretreatment of PLA2G1B (25 min at room temperature and 5 min at 37°C) with sPLA2 inhibitors (pentapeptide, varespladib, sPLA2R1) before a 15 min of stimulation with 2 nM IL-7 (recombinant glycosylated human IL-7, Accrobio System). All cell treatments were performed at 37°C. Cell supernatants were removed and stimulation was stopped by the addition of 500 µl of a 4% paraformaldehyde solution in PBS (Fisher, PFA 32% Electron Microscopy Science, 15714) and incubation for 15 min at 37 °C. Cells were then permeabilized overnight at -20°C in 500 µL of an ice-cold 90% methanol/water solution. Labelling of cells and Image analysis were performed as described in the Material and Methods section of the article.

PLA2 enzymatic assay on [3H] arachidonic acid-labeled CD4 T cells

To test sPLA2 inhibitors effect on PLA2G1B activity on [3H] arachidonic acid ([3H]-AA)-labeled CD4 T lymphocytes, cells were first thawed in 10% FBS RPMI preheated to 37°C, centrifuged at 580 x g for 10 min at room temperature, washed twice in 2.5% FBS RPMI, and equilibrated in 2.5% FBS RPMI at 2 x 10^5 CD4 T cells/400µL/well in 24-well polystyrene plates for 90 min at 37°C in a humidified 5% CO2 atmosphere. Then, 100 µL of recombinant WT human (hPLA2G1B), or medium or human PLA2G1B recombinant proteins, with a pretreatment (25 min at room temperature and 5 min at 37°C) with anti-PLA2G1B (14G9, previously described (1)) neutralizing mAb or control isotype (Mouse IgG1, 16-4714-85, Thermofisher), or sPLA2R1 or vehicle, in 2.5% FBS RPMI was added to each well and the plates incubated for 2 h.

To study PLA2G1B activity on [3H]-AA-labeled CD4 T lymphocytes relative to human proPLA2G1B, PLA2GIIA, PLA2GIID, PLA2GX, cells were prepared as above but equilibrated in 2.5% FBS RPMI at 10^6 CD4 T cells/400µL/well in 24-well polystyrene plates for 90 min at 37°C in a humidified 5% CO_2 atmosphere. Then, 100 µL of medium or recombinant sPLA2 proteins in 2.5% FBS RPMI was added to each well and the plates incubated for 2 h.

Cells and supernatants were collected in Eppendorf tubes and centrifuged at 580 x g for 10 min at room temperature. The [3H]-AA released into the cell supernatant was quantified in 300 µL using 16 mL of Ultima gold (Perkin Elmer, 6013329) in low diffusion vials (Perkin Elmer, 6000477) on a scintillation counter (tri-Carb 2800 TR liquid scintillation analyzer, Perkin Elmer).

To evaluate sPLA2 inhibitors effect, results are expressed as the percentage of inhibition of PLA2G1B activity on cells treated with PLA2G1B and several doses of inhibitors.

To evaluate PLA2G1B relative to other sPLA2 effect, results are shown as the percentage of PLA2 activity relative to that of PLA2G1B activity.

Supplementary Figures and Table

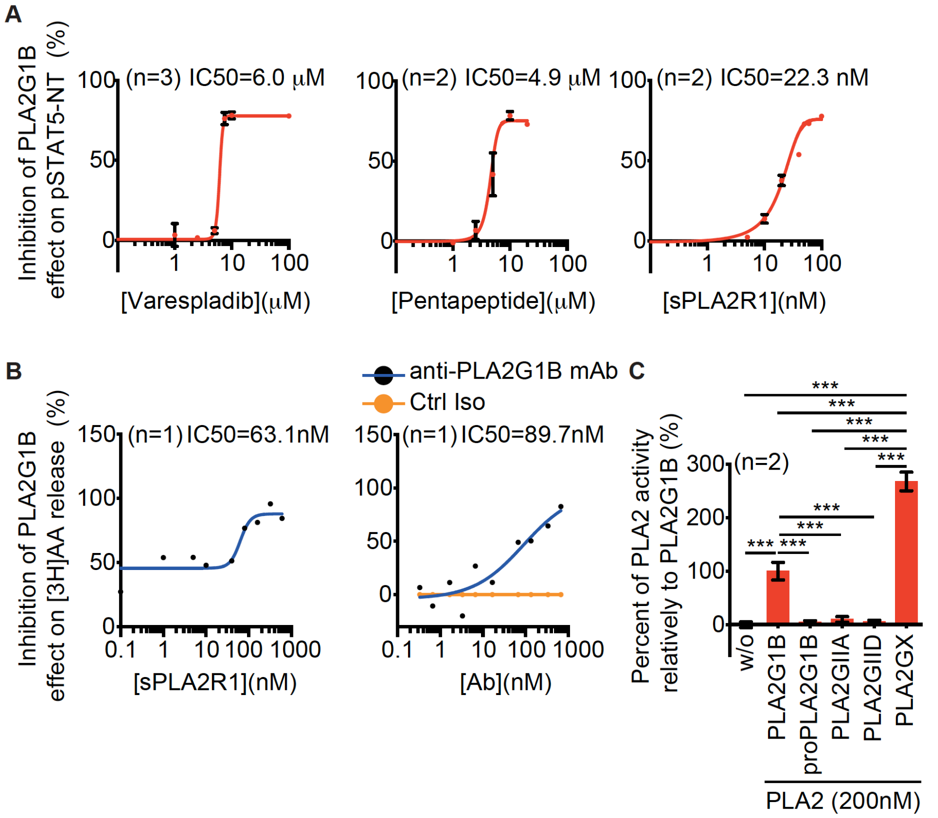

Supplementary Figure 1. PLA2G1B enzymatic activity on CD4 T-cell membranes correlates with inhibition of pSTAT5-NT response.

(**A**) Varespladib, pentapeptide, and sPLA2R1 treatment inhibits the effect of PLA2G1B on IL-7-induced pSTAT5-NT in a dose-dependent manner. Purified HD CD4 T cells were stimulated with IL-7, or not, and pSTAT5-nuclear translocation (pSTAT5-NT) was analyzed by confocal microscopy. Results are shown as the percentage of inhibition of PLA2G1B activity on cells treated with PLA2G1B (75 nM). IC50 values are presented. (**B**) sPLA2R1, and 14G9 anti-PLA2G1B mAb strongly inhibit PLA2G1B enzymatic activity on [3H]-AA-labeled CD4 T cells. Results are shown as the percentage of inhibition of PLA2G1B activity on cells treated with PLA2G1B (65 nM) and several doses of inhibitors. IC50 value are presented. (**C**) PLA2G1B and PLA2GX can digest CD4 T-cell membranes but not proPLA2G1B, PLA2GIIA or PLA2GIID. [3H]-AA-labeled CD4 T cells were seeded at 10^6 cells/well and treated in triplicate with several sPLA2 at 200 nM. Results are shown as the percentage of PLA2 activity relative to that of PLA2G1B activity. (A-C) Results are represented as the mean ± SD from a pool of n experiments as indicated. ****p* < 0.001 by two-way ANOVA with Tukey's correction for multiple comparisons.

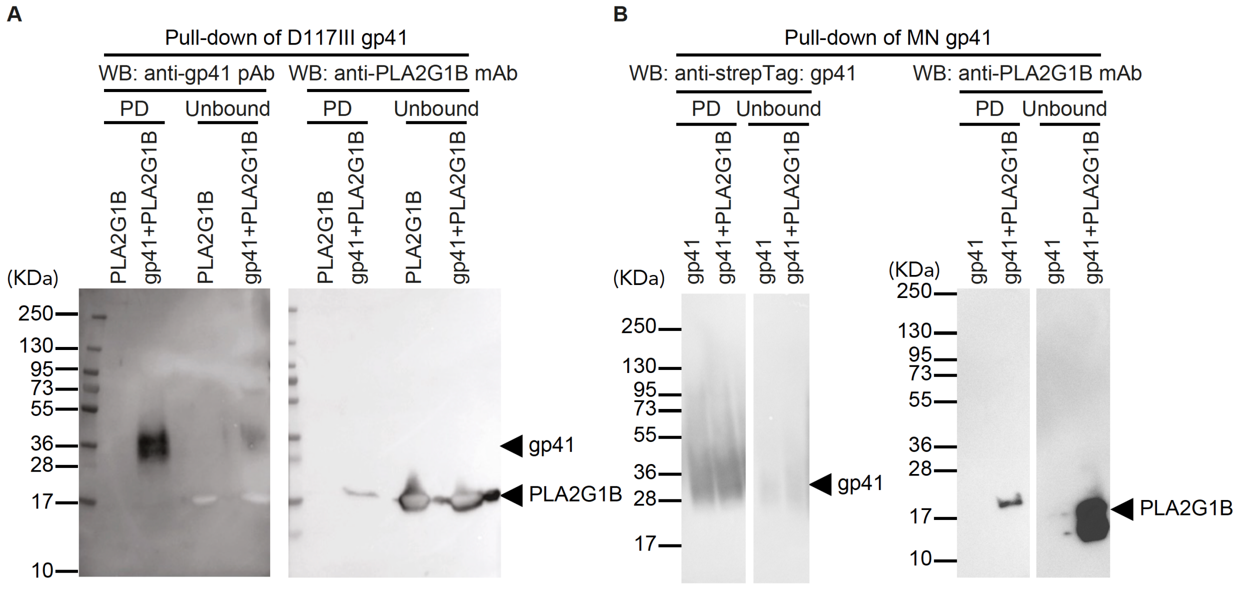

Supplementary Figure 2. PLA2G1B directly binds to D117III and MN gp41 in pull-down assays.

(**A**) Recombinant PLA2G1B protein (50 µg/mL) was incubated in PBS with strep-tagged-gp41 at 10 µg/mL (D117III strain, gp41+PLA2G1B) or not (PLA2G1B). (**B**) Recombinant strep-tagged gp41 (MN strain) at 5 µg/mL in PBS was incubated with recombinant PLA2G1B (50 µg/mL) (gp41+PLA2G1B) or not (gp41). (A, B) PLA2G1B-gp41 complexes were pulled-down with strep-Tactin XT-beads that pull-down gp41. Pulled-down products and unbound proteins were revealed by immunoblotting with anti-gp41 Ab (WB: goat polyclonal anti-gp41 Ab, anti-gp41 pAb in A, mouse anti-strep-tag mAb, anti-strepTag in B) or mouse anti-PLA2G1B mAb (WB: anti-PLA2G1B mAb). One representative experiment of five with similar results with D117III gp41 (A) and three with MN gp41 (B) are shown. The results in panel A represent the full western-blot data of the results presented in Figure 2E.

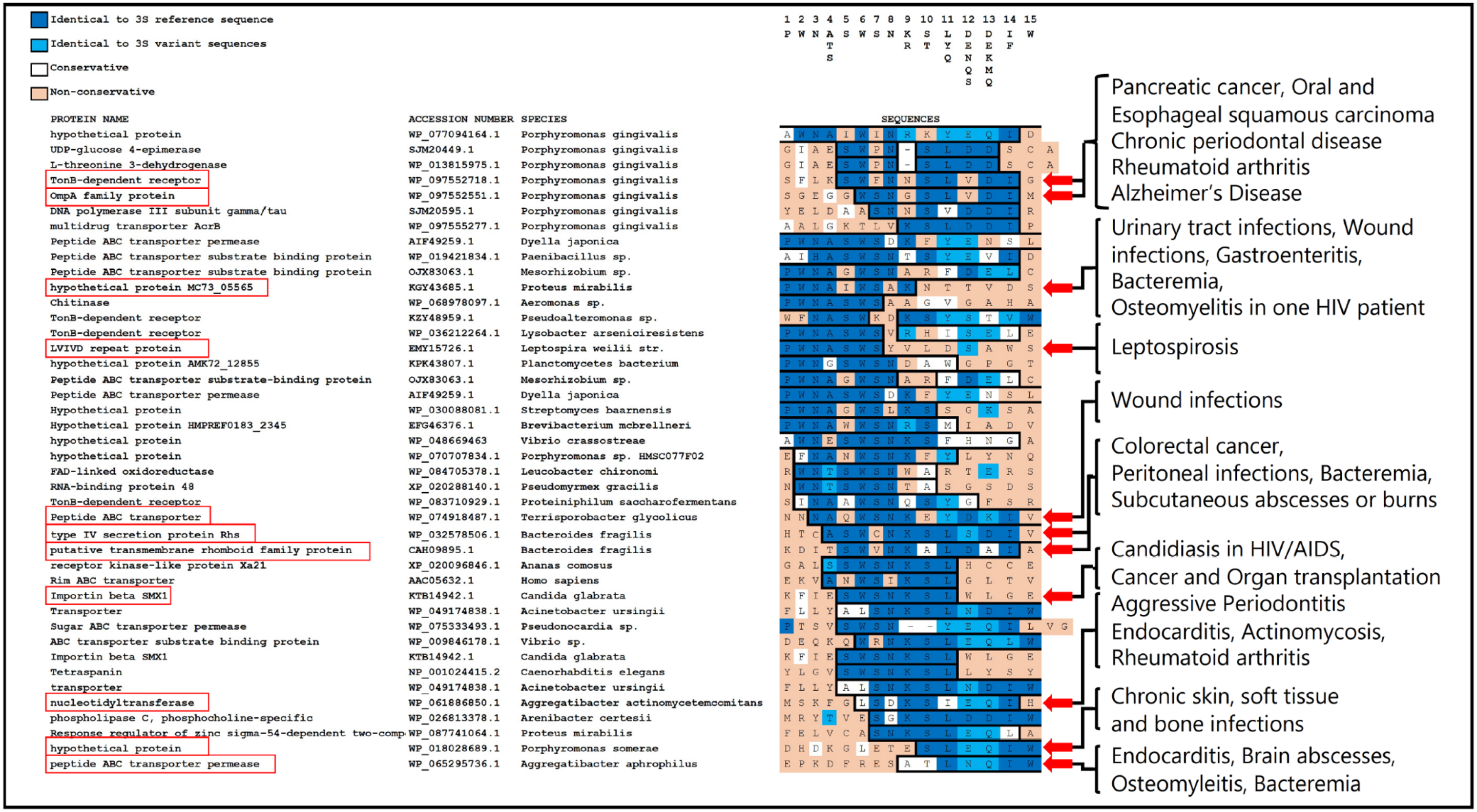

Supplementary Figure 3. Similarity search of 3S-like peptide motifs in sequence databases.

The amino-acid substitution matrix (AASM) shown at the top represents the original 3S peptide sequence PWNASWSNKSLDDIW, as previously described (1) with major amino-acid substitutions found in natural HIV-1 sequence variants. The 3S-AASM was used to search human and micro-organism protein sequences databases for similarity using Blastp with an EXPECT threshold of 100. The best sequence matches are presented in alignment with the 3S-AASM. The color scheme is defined as follows: dark blue background for amino-acid positions identical to the original 3S sequence, light blue background for amino-acid positions identical to 3S amino-acid variants, white background for conservative amino-acid positions sharing physico-chemical properties with the 3S-AASM, salmon pink background for non-conservative positions. Gaps were indicated by dashes (-). Human diseases associated with selected pathogen-derived peptides are indicated with a red arrow on the right-side with the corresponding protein name highlighted with a red box.

Supplementary Table 1. Selection of 3S-like peptides associated with human diseases.

ABBREVIATION	PROTEIN	SPECIES	SEQUENCES	PATHOLOGY
3S	gp41	HIV-1	PWNASWSNKSLDDIW	AIDS (1-6)
TonBdR Pg	TonB-dependent receptor	Porphyromonas gingivalis	SFLKSWFNNSLVDIG	Pancreatic cancer (7-13), Oral and Esophageal squamous carcinoma (9, 14-16), Chronic periodontal disease (7-13), Rheumatoid arthritis (17), Alzheimer's Disease (18)
OmpA Pg	OmpA family protein		SGEGGWSNGSLVDIM	
HP Pm	Hypothetical protein MC73_05565	Proteus mirabilis	PWNAIWSAKNTTVDS	Urinary tract infections, Wound infections, Gastroenteritis and Bacteremia (19-21), Osteomyelitis in one HIV patient (22)
LVIVD Lw	LVIVD repeat protein	Leptospira weilii str.	PWNASWSYVLDSAWS	Leptospirosis (23-26)
ABC Tg	Peptide ABC transporter	Terrisporobacter glycolicus	NNNAQWSNKEYDKIV	Wound infections (27)
Rhs Bf	Type IV secretion protein Rhs	Bacteroides fragilis	HTCASWCNKSLSDIV	Colorectal cancer (28-31), Peritoneal infections (32), Bacteremia (31, 33, 34), Subcutaneous abscesses or burns (35)
TM rhomboid Bf	Putative transmembrane rhomboid family protein		KDITSWVNKALDAIA	
bSMX1 Cg	Importin beta SMX1	Candida glabrata	KFIESWSNKSLWLGE	Candidiasis in HIV/AIDS (36, 37), Cancer (38-41) and Organ transplantation (42-45)
Nt trans Aa	nucleotidyltransferase	Aggregatibacter actinomycetemcomitans	MSKFGLSDKSIEQIH	Aggressive Periodontitis (13), Endocarditis (46-48), Actinomycosis (49, 50), Rheumatoid arthritis (17)
HP Ps	hypothetical protein	Porphyromonas somerae	DHDKGLETESLEQIW	Chronic skin, soft tissue and bone infections (51)
ABC Aa	peptide ABC transporter permease	Aggregatibacter aphrophilus	EPKDFRESATLNQIW	Endocarditis (46, 48), Brain abscesses (52, 53), Vertebral osteomyelitis (54), Bacteremia (55)

HIV-1 gp41 3S peptide sequence and information for selected peptides with 3S-like sequence are presented. Amino acids of the 3S motif SWSNKS are shown in red and those of the 3S peptide flanking the 3S motif in blue.

Supplementary data References

1. Vieillard V, Strominger JL, Debré P. NK cytotoxicity against CD4+ T cells during HIV-1 infection: a gp41 peptide induces the expression of an NKp44 ligand. *Proc Natl Acad Sci USA* (2005) 102:10981–6. doi:10.1073/pnas.0504315102

2. Fausther-Bovendo PH, Vieillard V, Sagan S, Bismuth G, Debré P. HIV gp41 engages gC1qR on CD4+ T cells to induce the expression of an NK ligand through the PIP3/H2O2 pathway. *PLoS Pathog* (2010) 6:1–14. doi:10.1371/journal.ppat.1000975

3. Vieillard V, Costagliola D, Simon A, Debré P, French Asymptomatiques à Long Terme (ALT) Study Group. Specific adaptive humoral response against a gp41 motif inhibits CD4 T-cell sensitivity to NK lysis during HIV-1 infection. *AIDS* (2006) 20:1795–804. doi:10.1097/01.aids.0000244198.65263.17

4. Petitdemange C, Achour A, Dispinseri S, Malet I, Sennepin A, Fang RHT, et al. A Single Amino-Acid Change in a Highly Conserved Motif of gp41 Elicits HIV-1 Neutralization and Protects Against CD4 Depletion. *Clin Infect Dis* (2013) 57:745–55. doi:10.1093/CID/CIT335

5. Pednekar L, Valentino A, Ji Y, Tumma N, Valentino C, Kadoor A, et al. Identification of the gC1qR sites for the HIV-1 viral envelope protein gp41 and the HCV core protein: Implications in viral-specific pathogenesis and therapy. *Mol Immunol* (2016) 74:18–26. doi:10.1016/J.MOLIMM.2016.03.016

6. Pothlichet J, Rose T, Bugault F, Jeammet L, Meola A, Haouz A, et al. PLA2G1B is involved in CD4 anergy and CD4 lymphopenia in HIV-infected patients. *J Clin Invest* (2020) 130:2872–87. doi:10.1172/JCI131842

7. Gnanasekaran J, Gallimidi AB, Saba E, Pandi K, Berchoer LE, Hermano E, et al. Intracellular Porphyromonas gingivalis promotes the tumorigenic behavior of pancreatic carcinoma cells. *Cancers (Basel)* (2020) 12:1–14. doi:10.3390/cancers12082331

8. Liu XB, Gao ZY, Sun CT, Wen H, Gao B, Li SB, et al. The potential role of P.gingivalis in gastrointestinal cancer: A mini review. *Infect Agent Cancer* (2019) 14: doi:10.1186/s13027-019-0239-4

9. Olsen I, Yilmaz Ö. Possible role of Porphyromonas gingivalis in orodigestive cancers. *J Oral Microbiol* (2019) 11: doi:10.1080/20002297.2018.1563410

10. Fan X, Alekseyenko A v., Wu J, Peters BA, Jacobs EJ, Gapstur SM, et al. Human oral microbiome and prospective risk for pancreatic cancer: A population-based nested case-control study. *Gut* (2018) 67:120–7. doi:10.1136/gutjnl-2016-312580

11. Torres PJ, Fletcher EM, Gibbons SM, Bouvet M, Doran KS, Kelley ST. Characterization of the salivary microbiome in patients with pancreatic cancer. *PeerJ* (2015) 2015: doi:10.7717/peerj.1373

12. Michaud DS. Role of bacterial infections in pancreatic cancer. *Carcinogenesis* (2013) 34:2193–2197. doi:10.1093/carcin/bgt249

13. Michaud DS, Izard J, Wilhelm-Benartzi CS, You DH, Grote VA, Tjønneland A, et al. Plasma antibodies to oral bacteria and risk of pancreatic cancer in a large European prospective cohort study. *Gut* (2013) 62:1764–70. doi:10.1136/gutjnl-2012-303006

14. Chen MF, Lu MS, Hsieh CC, Chen WC. Porphyromonas gingivalis promotes tumor progression in esophageal squamous cell carcinoma. *Cell Oncol (Dordr)* (2021) 44:373–84. doi:10.1007/S13402-020-00573-X

15. Kong J, Yuan X, Wang J, Liu Y, Sun W, Gu B, et al. Frequencies of Porphyromonas gingivalis Detection in Oral-Digestive Tract Tumors. *Pathol Oncol Res* (2021) 27: doi:10.3389/pore.2021.628942

16. Qi YJ, Jiao YL, Chen P, Kong JY, Gu BL, Liu K, et al. Porphyromonas gingivalis promotes progression of esophageal squamous cell cancer via TGFβ-dependent Smad/YAP/TAZ signaling. *PLoS Biol* (2020) 18: doi:10.1371/JOURNAL.PBIO.3000825

17. Gómez-Bañuelos E, Mukherjee A, Darrah E, Andrade F. Rheumatoid Arthritis-Associated Mechanisms of Porphyromonas gingivalis and Aggregatibacter actinomycetemcomitans. *J Clin Med* (2019) 8:1309. doi:10.3390/jcm8091309

18. Dominy SS, Lynch C, Ermini F, Benedyk M, Marczyk A, Konradi A, et al. Porphyromonas gingivalis in Alzheimer's disease brains: Evidence for disease causation and treatment with small-molecule inhibitors. *Sci Adv* (2019) 5: doi:10.1126/sciadv.aau3333

19. Mobley HLT. Proteus mirabilis Overview. *Methods Mol Biol* (2019) 2021:1–4. doi:10.1007/978-1-4939-9601-8_1

20. Armbruster CE, Mobley HLT, Pearson MM. Pathogenesis of Proteus mirabilis Infection. *EcoSal Plus* (2018) 8: doi:10.1128/ECOSALPLUS.ESP-0009-2017

21. Schaffer JN, Pearson MM. Proteus mirabilis and Urinary Tract Infections. *Microbiol Spectr* (2015) 3: doi:10.1128/MICROBIOLSPEC.UTI-0017-2013

22. Petsatodis G, Symeonidis PD, Karataglis D, Pournaras J. Multifocal Proteus mirabilis osteomyelitis requiring bilateral amputation in an HIV-positive patient. *J Bone Joint Surg Br* (2007) 89:249–51. doi:10.1302/0301-620X.89B2.18167

23. Jayasundara D, Senavirathna I, Warnasekara J, Gamage C, Siribaddana S, Kularatne SAM, et al. 12 novel clonal groups of leptospira infecting humans in multiple contrasting epidemiological contexts in Sri Lanka. *PLoS Negl Trop Dis* (2021) 15: doi:10.1371/journal.pntd.0009272

24. Slack AT, Symonds ML, Dohnt MF, Corney BG, Smythe LD. Epidemiology of Leptospira weilii serovar Topaz infections in Australia. *Commun Dis Intell Q Rep* (2007) 31:216–22. Available at: https://pubmed.ncbi.nlm.nih.gov/17724998/

25. Xu Y, Zheng H, Zhang Y, Wang Y, Zhang J, Li Z, et al. Genomic analysis of a new serovar of Leptospira weilii serogroup Manhao. *Front Microbiol* (2017) 8: doi:10.3389/FMICB.2017.00149

26. Mason MR, Encina C, Sreevatsan S, Muñoz-Zanzi C. Distribution and Diversity of Pathogenic Leptospira Species in Peri-domestic Surface Waters from South Central Chile. *PLoS Negl Trop Dis* (2016) 10: doi:10.1371/journal.pntd.0004895

27. Cheng MP, Domingo MC, Lévesque S, Yansouni CP. A case report of a deep surgical site infection with Terrisporobacter glycolicus/T. Mayombei and review of the literature. *BMC Infect Dis* (2016) 16: doi:10.1186/S12879-016-1865-8

28. Cheng Y, Ling Z, Li L. The Intestinal Microbiota and Colorectal Cancer. *Front Immunol* (2020) 11: doi:10.3389/fimmu.2020.615056

29. Chung L, Thiele Orberg E, Geis AL, Chan JL, Fu K, DeStefano Shields CE, et al. Bacteroides fragilis Toxin Coordinates a Pro-carcinogenic Inflammatory Cascade via Targeting of Colonic Epithelial Cells. *Cell Host Microbe* (2018) 23:203-14.e5. doi:10.1016/j.chom.2018.01.007

30. Sears CL, Geis AL, Housseau F. Bacteroides fragilis subverts mucosal biology: From symbiont to colon carcinogenesis. *J Clin Invest* (2014) 124:4166–72. doi:10.1172/JCI72334

31. Kwong TNY, Wang X, Nakatsu G, Chow TC, Tipoe T, Dai RZW, et al. Association Between Bacteremia From Specific Microbes and Subsequent Diagnosis of Colorectal Cancer. *Gastroenterology* (2018) 155:383-90.e8. doi:10.1053/j.gastro.2018.04.028

32. Chao C ter, Lee SY, Yang WS, Chen HW, Fang CC, Yen CJ, et al. Peritoneal dialysis peritonitis by anaerobic pathogens: A retrospective case series. *BMC Nephrol* (2013) 14: doi:10.1186/1471-2369-14-111

33. Brook I. The role of anaerobic bacteria in bacteremia. *Anaerobe* (2010) 16:183–9. doi:10.1016/j.anaerobe.2009.12.001

34. Brook I. Microbiology and management of abdominal infections. *Dig Dis Sci* (2008) 53:2585–91. doi:10.1007/s10620-007-0194-6

35. Brook I, Coolbaugh JC, Walker RI. Antibiotic and clavulanic acid treatment of subcutaneous abscesses caused by bacteroides fragilis alone or in combination with aerobic bacteria. *J Infect Dis* (1983) 148:156–9. doi:10.1093/infdis/148.1.156

36. Demiraslan H, Alabay S, Ulu-Kilic A, Borlu M, Doganay M. Cutaneous candidiasis caused by Candida glabrata in a HIV/AIDS patient. *Int J STD AIDS* (2013) 24:753–5. doi:10.1177/0956462413479897

37. Mushi MF, Bader O, Taverne-Ghadwal L, Bii C, Groß U, Mshana SE. Oral candidiasis among African human immunodeficiency virus-infected individuals: 10 years of systematic review and meta-analysis from sub-Saharan Africa. *J Oral Microbiol* (2017) 9: doi:10.1080/20002297.2017.1317579

38. Farmakiotis D, Tarrand JJ, Kontoyiannis DP. Drug-Resistant candida glabrata infection in cancer patients. *Emerg Infect Dis* (2014) 20:1833–40. doi:10.3201/EID2011.140685

39. Hachem R, Hanna H, Kontoyiannis D, Jiang Y, Raad I. The changing epidemiology of invasive candidiasis: Candida glabrata and candida krusei as the leading causes of candidemia in hematologic malignancy. *Cancer* (2008) 112:2493–9. doi:10.1002/CNCR.23466

40. Li D, Li T, Bai C, Zhang Q, Li Z, Li X. A predictive nomogram for mortality of cancer patients with invasive candidiasis: a 10-year study in a cancer center of North China. *BMC Infect Dis* (2021) 21: doi:10.1186/S12879-021-05780-X

41. Miceli MH, Díaz JA, Lee SA. Emerging opportunistic yeast infections. *The Lancet Infect Dis* (2011) 11:142–51.

42. Schwartz IS, Patterson TF. The Emerging Threat of Antifungal Resistance in Transplant Infectious Diseases. *Curr Infect Dis Rep* (2018) 20: doi:10.1007/S11908-018-0608-Y

43. Fernández-Ruiz M, Cardozo C, Salavert M, Aguilar-Guisado M, Escolà-Vergé L, Muñoz P, et al. Candidemia in solid organ transplant recipients in Spain: Epidemiological trends and determinants of outcome. *Transpl Infect Dis* (2019) 21: doi:10.1111/TID.13195

44. Andes DR, Safdar N, Baddley JW, Alexander B, Brumble L, Freifeld A, et al. The epidemiology and outcomes of invasive Candida infections among organ transplant recipients in the United States: results of the Transplant-Associated Infection Surveillance Network (TRANSNET). *Transpl Infect Dis* (2016) 18:921–31. doi:10.1111/TID.12613

45. Zarei F, Hashemi SJ, Salehi M, Mahmoudi S, Zibafar E, Ahmadinejad Z, et al. Molecular characterization of fungi causing colonization and infection in organ transplant recipients: A one-year prospective study. *Curr Med Mycol* (2020) 6:30–5. doi:10.18502/CMM.6.1.2505

46. Revest M, Egmann G, Cattoir V, Tattevin P. HACEK endocarditis: state-of-the-art. *Expert Rev Anti Infect Ther* (2016) 14:523–30. doi:10.1586/14787210.2016.1164032

47. Sharara SL, Tayyar R, Kanafani ZA, Kanj SS. HACEK endocarditis: a review. *Expert Rev Anti Infect Ther* (2016) 14:539–45. doi:10.1080/14787210.2016.1184085

48. Berge A, Morenius C, Petropoulos A, Nilson B, Rasmussen M. Epidemiology, bacteriology, and clinical characteristics of HACEK bacteremia and endocarditis: a population-based retrospective study. *Eur J Clin Microbiol Infect Dis* (2021) 40:525–34. doi:10.1007/S10096-020-04035-Y

49. Sharma S, Hashmi MF, Valentino III DJ. Actinomycosis. *StatPearls* (2021) Available at: http://www.ncbi.nlm.nih.gov/pubmed/29489261

50. Kuijper EJ, Wiggerts HO, Jonker GJ, Schaal KP, Gans J de. Disseminated actinomycosis due to actinomyces meyeri and actinobacillus actinomycetemcomitans. *Scand J Infect Dis* (1992) 24:667–72. doi:10.3109/00365549209054655

51. Summanen PH, Durmaz B, Väisänen ML, Liu C, Molitoris D, Eerola E, et al. Porphyromonas somerae sp. nov., a pathogen isolated from humans and distinct from Porphyromonas levii. *J Clin Microbiol* (2005) 43:4455–9. doi:10.1128/JCM.43.9.4455-4459.2005

52. Miller DC, Fung M, Carbo A. A Furry Friend's Dirty Mouth: Brain Abscess Due to Aggregatibacter (Haemophilus) aphrophilus. *Am J Med* (2017) 130:e447–e448. doi:10.1016/J.AMJMED.2017.04.020

53. Maraki S, Papadakis IS, Chronakis E, Panagopoulos D, Vakis A. Aggregatibacter aphrophilus brain abscess secondary to primary tooth extraction: Case report and literature review. *J Microbiol Immunol Infect* (2016) 49:119–22. doi:10.1016/J.JMII.2013.12.007

54. Khurana A, Kowlgi NG, Naut ER. Retroperitoneal, psoas, and scrotal abscesses due to an uncommon organism - Aggregatibacter aphrophilus: Case report and review of literature. *Conn Med* (2016) 80:543–7.

55. Chien YC, Huang YT, Liao CH, Chien JY, Hsueh PR. Clinical characteristics of bacteremia caused by Haemophilus and Aggregatibacter species and antimicrobial susceptibilities of the isolates. *J Microbiol Immunol Infect* (2020) doi:10.1016/J.JMII.2020.12.002
